# Differential expression of peptidases in *Strigomonas culicis* wild-type and aposymbiotic strains: from proteomic data to proteolytic activity

**DOI:** 10.1590/0074-02760240110

**Published:** 2024-12-09

**Authors:** Julia Fernandes Barbosa dos Santos, Ana Cristina Souza Bombaça, Bianca da Silva Vitório, Geovane Dias-Lopes, Aline dos Santos Garcia-Gomes, Rubem Sadok Figueiredo Menna-Barreto, Claudia Masini d’Avila, Vítor Ennes-Vidal

**Affiliations:** 1 Fundação Oswaldo Cruz-Fiocruz, Instituto Oswaldo Cruz, Laboratório de Doenças Parasitárias, Rio de Janeiro, RJ, Brasil; 2 Fundação Oswaldo Cruz-Fiocruz, Instituto Oswaldo Cruz, Laboratório de Biologia Celular, Rio de Janeiro, RJ, Brasil; 3 Fundação Oswaldo Cruz-Fiocruz, Instituto Oswaldo Cruz, Laboratório de Biologia Molecular e Doenças Endêmicas, Rio de Janeiro, RJ, Brasil; 4 Universidade do Estado do Rio de Janeiro, Instituto de Biologia Roberto Alcântara Gomes, Departamento de Ciências Biomédicas e Saúde, Cabo Frio, RJ, Brasil; 5 Instituto Federal de Educação, Ciência e Tecnologia do Rio de Janeiro, Laboratório de Microbiologia, Rio de Janeiro, RJ, Brasil

**Keywords:** trypanosomatids, proteome, proteolysis, GP63, calpains

## Abstract

**BACKGROUND:**

*Strigomonas culicis* is a monoxenic trypanosomatid parasite of insects that naturally contains an endosymbiotic bacterium. The aposymbiotic strain can be obtained, making this strain a model for evolutive research about organelle origins. In addition, *S. culicis* contains homologues of virulence factors of pathogenic trypanosomatids, which functions are waiting for further analysis. In this sense, the publication of *S. culicis* proteome makes feasible additional investigations regarding the differential expression of peptidases from the wild-type (WT) and the aposymbiotic (APO) strains.

**OBJECTIVES:**

Here, we analysed two proteomic data from *S. culicis* WT and APO strains screening for peptidases differentially expressed and assessed the differential expression of cysteine and metallopeptidases.

**METHODS:**

A comparative proteomic screening between WT and APO identified 43 modulated peptidases.

**FINDINGS:**

Cysteine and metallopeptidases, such as calpains and GP63, were the major classes, highlighting their significance. GP63 exhibited an increased proteolysis in a specific metallopeptidase substrate, an up-modulation gene expression in RT-PCR, and a higher protein identification by flow cytometry in the aposymbiotic strain. Notwithstanding, the wild-type strain showed enhanced cysteine peptidase activity.

**MAIN CONCLUSION:**

Our study highlighted the endosymbiont influence in *S. culicis* peptidase expression, with GP63 expression and activity raised in the aposymbiotic strain, whereas cysteine peptidase levels were reduced.

The Trypanosomatidae family (Euglenozoa; Kinetoplastea) comprises a group of obligatory protozoa parasites, grouped into heteroxenous and monoxenous species. Heteroxenic trypanosomatids have complex life cycles involving multiple hosts, responsible for human diseases like Chagas’ disease (*Trypanosoma cruzi*), sleeping sickness (*Trypanosoma brucei*), and leishmaniasis (*Leishmania* spp.). Unfortunately, the current treatment of such neglected tropical diseases is limited to drugs that suffer from unacceptable toxicity, high costs, difficulties of administration and increasing treatment failures due to the parasite resistance.^1^

Monoxenous species, which infects *a priori* only insects, are much less studied. However, these protozoa could be used as a model for studying host-parasite interactions, evolutionary adaptations, and therapeutic strategies.^2^ ^,^ ^3^ These organisms also represent alternatives for vaccine candidates, for a platform of eukaryotic protein expression, or even as a biological control for insect vectors.^4^ Moreover, monoxenic trypanosomatids are attracting the attention due to the increasing reports on the presence of these presumably non-pathogenic trypanosomatids in humans.^4^ ^,^ ^5^

Within the monoxenic trypanosomatids, the Strigomonadinae subfamily ― including the genera *Strigomonas*, *Angomonas*, and *Kentomonas* ― stands out for its mutualistic relationship with *Candidatus* Kinetoplastibacterium spp.^6^ ^,^ ^7^ This symbiotic association provides essential nutrients and metabolic precursors critical for the growth and viability of the host trypanosomatids.^6^ ^,^ ^8^ The possibility of elimination of the bacteria provides an aposymbiotic (APO) strain, making feasible comparative studies with the wild-type (WT) strain. Some of these studies revealed the endosymbiont’s involvement on the metabolism of amino acids, vitamins, cofactors, lipids, and nucleotides, impairing the protein synthesis, energy production, and others metabolic pathways.^9^

Recently, two proteomic analyses compared WT and APO strains of *Srigomonas culicis*, revealing significant impacts of endosymbiont absence on amino acid synthesis and protein folding.^10^ ^,^ ^11^ However, functional validations are required to confirm these omics findings. In this sense, peptidases are promising target that deserve more studies. These enzymes are responsible for cleaving peptide bonds and are implicated in both normal and disease-related processes.^1^ Consequently, the peptidases emerged as potential new drug target to treat infectious diseases, such as caused by trypanosomatids.^1^

The publication of the *S. culicis* whole genome enables the identification of peptidases poorly expressed or biochemically difficult to detect.^9^ Therefore, to assess the symbiont’s influence of peptidases on metabolic pathways, we compared the previous *S. culicis* proteomic data analysing the differential expression between WT and APO strains. The most relevant targets were selected for relative gene expression analysis. In addition, enzymatic assays with substrates from different peptidases’ classes were performed to compare the proteolytic activity of both strains. Finally, polyclonal antibodies raised against calpain and GP63 were used to evaluate these protein expression levels.

## MATERIALS AND METHODS

*Peptidase search in S. culicis genome, proteomes, and Gene Ontology* - Peptidase sequences of *S. culicis* (Genome ID 14536) were retrieved from *GenBank* database (www.ncbi.nlm.nih.gov/genome). These proteins were analysed for InterPro families and domains databases by Interproscan v5.51‐85.0 to confirm the presence of peptidases domains. Additionally, molecular mass predictions were performed using the “Protein Molecular Weight” computational tool (www.bioinformatics.org/sms/prot_mw.html.^12^ Simultaneously, *S. culicis* peptidases were searched in MEROPS.

Proteomic data obtained from the WT and APO strains of two proteomes^10^ ^,^ ^11^ were analysed as two datasets. The identified peptidases were categorised according to Gene Ontology (GO) categories available in UniProt, as follow: (i) cellular component; (ii) biological process; and (iii) molecular function. To elucidate metabolic pathways, the KEGG metabolic pathways database (www.genome.jp/kegg) was used employing *Leishmania major* protein codes (T number: T01014).

*Parasites cultivation* - The WT and APO strains were obtained from the Coleção de Protozoários (COLPROT) - FIOCRUZ. The axenic cultures were established through twice-weekly subcultures in liver infusion and tryptosis (LIT) culture medium pH 7.2, supplemented with 0,1% (w/v) hemin and 10% (WT) or 20% (APO) calf bovine serum. For DNA and peptidase extraction, 1.0 x 10⁹ cells were harvested by centrifugation in PBS (phosphate buffered saline, 150 mM NaCl, 20 mM phosphate buffer, pH 7.2).

*Gene expression comparison between WT and APO strains* - The peptidases identified as differentially expressed were selected for gene-specific primer design using Primer3Plus (www.bioinformatics.nl/cgi-bin/primer3plus/primer3plus.cgi) [Supplementary data (Table I)]. The primers were validated by conventional polymerase chain reaction (PCR) to confirm the amplification of a single product. Subsequently, the mRNA previously isolated using the TRIzol^®^ reagent (Invitrogen) was treated with DNAse I (Sigma-Aldrich) and submitted to reverse with the SuperScriptIII kit (Applied Biosystems). For qPCR, diluted cDNA (~ 80 ng/μL) was used in 20 µL reaction with “SYBR Green q PCR Master Mix” reagent according to the manufacturer’s instructions. Real-time reactions were performed on an ABI Prism 7500 FAST system (Applied Biosystems).^13^ The relative gene expression was determined using comparative Ct values, and the relative expression was reported as 2^−ΔΔCt^, with error bars indicating the standard deviation of the mean.^14^ Actin and paraflagellar rod genes were used as endogenous control.

*Peptidase enzymatic assays* - To obtain protein extracts, cells were lysed using 2% CHAPS (Sigma), and the soluble and insoluble fractions were separated by centrifugation at 14,000 × *g* at 4ºC. The soluble fraction was quantified using the Pierce™ BCA Protein Assay Kit (Thermo Fischer) and stored at -80ºC. The proteolytic activity of WT and APO strains were analysed by zymography and solution-based assays. For zymography analysis, 40 μg of protein from each sample were loaded onto 12% polyacrylamide gels co-polymerised with either 0.1% gelatine or 0.1% casein. Electrophoresis was conducted in an ice bath under non-reducing conditions with a constant voltage of 120 V. Subsequently, the gels were incubated with 5 mM DTT in the following reaction buffers: 50 mM phosphate buffer (pH 5.5) or 100 mM Tris-HCl (pH 7.4), at 37ºC for 72 h. Staining was performed using Coomassie Blue R-250 in methanol-acetic acid-water, and destained in the same solvent. Additionally, incubation was conducted with proteolytic inhibitors, including 10 mM of 1,10-phenanthroline (PHE) and ethylenediaminetetraacetic acid (EDTA), 10 μM of trans-epoxysuccinyl-L-leucylamido-(4-guanidino)butane (E-64) and 1 mM of phenylmethylsulfonyl fluoride (PSMF).^12^

For in-solution assays, enzymatic activity was continuously evaluated at 37ºC through the incubation of protein extracts with four different fluorogenic peptide substrates. For broad-range activity, fluorescein-labelled DQ gelatine conjugate (Gelatine-FITC) was assessed in 0.5 M Tris-HCL, pH 7.6; for cysteine peptidase activity, Z-carbobenzoxy-L-phenylalanyl-L-arginine-7-amino-4-methylcoumarin (Z-Phe-Arg-AMC) was utilised in sodium phosphate buffer, pH 5.5;^15^ for metallopeptidase activity, MCA‐Pro‐Cha‐Gly‐Nva‐His‐Ala‐Dpa‐NH2 (Matrix metallopeptidase 13- MMP13) (Sigma) was evaluated in glycine‐NaOH buffer, pH 10.0 containing 1 mM CaCl_2_ and 1 mM dithiothreitol (DTT);^12^ and the proteasome activity was observed with Z-Leu-Leu-Leu-7-amido-4-methylcoumarin (Z-LLL-AMC) (Sigma) in 500 mM Tris-HCl buffer, pH 7.4 containing 5 mM MgCl_2_, 1 mM DTT and 1 mM ATP.^16^ The inhibition experiments were conducted through incubation with a range of inhibitors, including 10 mM PHE, ethyleneglycol-bis(β-aminoethyl)-N,N,Nʹ,Nʹ-tetraacetic acid (EGTA), and EDTA, 10 μM E-64, 1 mM PSMF, and 20 μM lactacystin. The assays were performed under light shielded conditions in black flat bottom 96‐microwell plates employing the Spectra Max Gemini spectrofluorometer (Molecular Devices), with excitation and emission wavelengths accordingly with manufacturer’s instructions. The experiments were monitored for the spontaneous release of the fluorophore during 1 hour with 30-second intervals. All findings were replicated three times in triplicate and are presented as mean ± standard deviation.

*Flow cytometry and western blotting analysis* - The differential protein-level of identified peptidases was assessed through the reactivity of specific antibodies by western blotting and flow cytometry. For flow cytometry analysis, cells from both *S. culicis* strains were fixed in 4% paraformaldehyde at room temperature. Subsequently, cells were incubated with primary antibodies anti-GP63 (1:1,000) and anti-tritryp-calpain (1:250) for 2 h, followed by incubation with secondary antibody anti-IgG conjugated to Alexa-488 fluorophore at 1:750 dilution. The omission of primary antibody was employed as control. Approximately 10,000 parasites from each condition were quantified in triplicate using the FACS Calibur equipment (BD Bioscience).

For blotting, nitrocellulose membranes containing the transferred WT and APO proteins were blocked in 10% low-fat dried milk dissolved in PBS containing 2% Tween 20 (TBS/Tween) overnight at 4ºC. The membranes were then washed with the blocking solution and subsequently incubated for 2 h with primary antibodies anti-GP63 at 1:2,000 dilution, or anti-tritryp-calpain at 1:1,000 dilution. After additional washes, the membranes were incubated for 1 h with secondary antibody anti-IgG conjugated to peroxidase at 1:2,000 dilution. Finally, the membranes were subjected to chemiluminescence detection. Image acquisition was performed using the Image Quant 400 equipment (GE Healthcare).^13^ An anti-β-actin polyclonal antibody (Rhea Biotech) (1:5,000) was used as a loading control. The reactive polypeptides molecular masses were calculated by comparing with the mobility of the sodium dodecyl-sulfate polyacrylamide gel electrophoresis (SDS-PAGE) standards, and the densitometric analysis was performed using the ImageJ.

*Statistical analysis* - All experiments were repeated at least three times with triplicate samples, and the results were expressed as the mean and standard deviation. Statistical analysis was performed using GraphPad Prism Version 5.00 software, utilising 2-way ANOVA, Mann-Whitney, or Student’s *t* tests. A significance level of p < 0.05 was applied to determine statistical significance.

## RESULTS AND DISCUSSION

*Expansion of calpain and GP63 sequences in S. culicis genome* - Peptidases comprise approximately 2% of total proteins in all organisms, playing essential roles in cellular functions. These enzymes are classified based on the key amino acid for the catalytic mechanism as serine, cysteine, threonine, aspartic, glutamic, or metallo types.^17^ In trypanosomatids, peptidases are considered as virulence factors and potential drug targets.^1^ Their presence in monoxenic organisms highlights its nourishment significance and involvement in the interaction process with invertebrate host cells.^18^ In this sense, the *S. culicis* genome published by Motta and co-workers^9^ provided a valuable source to identify homologous of peptidases from pathogenic organisms, as well as to find proteolytic enzymes not yet described in that organism.

Here, peptidases were identified in *S. culicis* genome querying the NCBI database, revealing a total of 334 sequences. The most abundant categories retrieved were, respectively metallo (119), cysteine (114), threonine (46), serine (25), and aspartic peptidases (27). Remarkably, 23 sequences lacked categorisation of class and domain (Table I). Two of the most abundant identified peptidases were calpains and GP63, presenting 49 and 8 copies, respectively [Supplementary data (Table II)]. Divergent results were reported from the genome study,^9^ where 62 calpains and nine GP63 were found. Possible explanations for these discrepancies include: (i) variations in annotation or updates in genome databases; or (ii) inclusion of hypothetical proteins (50 sequences), lacking defined peptidase’s classes. Simultaneous investigations in MEROPS database revealed 114 peptidases from *S. culicis*.^17^ The divergence of 194 identified sequences in MERPOS were probably in accordance with the same inconsistencies reported by the NCBI analysis. These sequences were classified as cysteine (56), metallo (52), threonine (18), serine (11), and aspartic peptidases (3), respectively (Table I), including 26 calpains and 8 GP63 sequences.

**TABLE I:** *Strigomonas culicis* peptidases identified in *GenBank* and MEROPS and their proteolytic classes (Genome ID 14536)

Peptidases	NCBI (GenBank)	MEROPS
Metallo	119	52
Cysteine	114*	56
Threonine	46	18
Serine	25	11
Aspartic	7	3
No defined class	23	0
Total	334	140

* No catalytic domain was identified in 17 sequences

Similar expansion of calpains and GP63 sequences were observed in heteroxenic trypanosomatids, such as *T. cruzi, T. brucei* and *Leishmania braziliensis*, which present 63, 25 and 34 calpain sequences, respectively;^19^ and *Leishmania tarentolae*, with 61 GP63 annotations.^20^ A potential conserved role or evolutionary pressure could lead the expansion of calpains and GP63 across these species. The reference for the calpain classification corresponds to the mammalian conventional calpain domain organisation, which correspond to the “classical” calpains, in contrast to “non-classical” ones that differ in their domain arrangement.^21^ However, the classification of calpains in families is constantly being updated. Trypanosomatids harbour a large and diverse family of calpain sequences in their genomes, comprising a wide range of associated domains, differential gene expression among life-cycle forms, and ubiquitous distribution in the parasite cell body.^19^ In *S. culicis*, significant disparities arise, such as the lack of the calpain-type beta-sandwich domain (CBSW). In contrast, GP63 sequences in *S. culicis* and *L. tarentolae* presented consistent features, such as the presence of the metallopeptidase M8 as the unique domain, zinc biding motifs, transmembrane helices, and similar molecular weight (data not shown).

*Peptidases are differentially expressed between S. culicis WT and APO strains* - Although genomic studies add consistent data about an organism’s genetic composition, proteomic analysis could explore practical implications of coding genes, providing a more detailed understanding of protein function and expression dynamics. Expanding this idea, our research group conducted two comparative proteomic analyses between the WT and APO strains of *S. culicis*. Brunoro and co-workers^10^ studied the intricate metabolic relationship within the symbiotic association, while Bombaça’s proteome^11^ focused on elucidating the effects of oxidative stress and metabolic alterations, employing an additional wild-type ROS-resistant strain (WTR). Comparisons between APO and WT strains disclosure changes across several metabolic pathways. The APO strain exhibited increased glycerol secretion, indicating a shift to fermentative metabolism and decreased energy efficiency without the symbiont.^22^ Additionally, changes in oxidative phosphorylation and amino acid metabolism were observed, along with alterations in carbohydrate breakdown and ATP production. Increased oxygen consumption and ATP release suggest an intensified electron transport system (ETS) activity in the presence of symbionts, as occurs in *S. culicis*. Moreover, variations in glutathione metabolism highlighted the dynamic interaction between metabolic pathways and environmental stressors.^8^ ^,^ ^10^ ^,^ ^11^

**Fig. 1:**
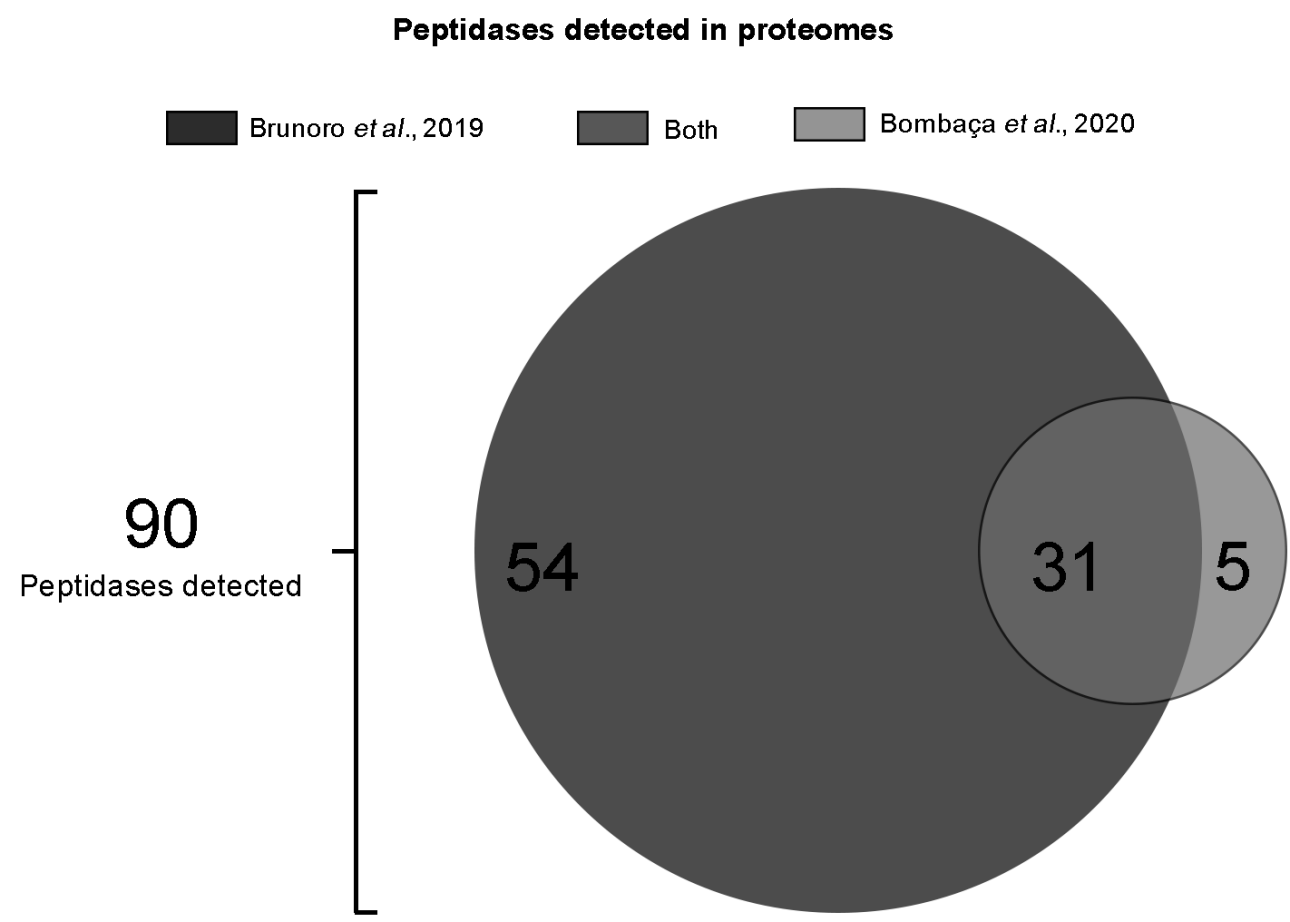
venn diagram of the peptidases found in proteomic data from *Strigomonas culicis*. Of 90 peptidases, 54 peptidases were exclusively detected in the Brunoro’s proteome^10^ (dark grey), 31 were detected in both proteomic surveys (medium grey) and five were detected in Bombaça’s proteome^11^ (light grey).

Given the significant metabolic alterations observed in the proteomic analyses, this study aimed to investigate the role of peptidases regulating cellular processes in *S. culicis*. Therefore, 90 peptidases were identified in both the WT and APO strains within the two published proteomes (Fig. 1). It is noteworthy to mention that the study by Brunoro^10^ encompassed three specific time-points of the parasite growth combined in a single dataset, while Bombaça’s proteome^11^ focused on the log phase, resulting in a different number of detected peptidases between the studies. Remarkably, metallo and cysteine peptidases were the major classes, as already reported by genomic screens in other trypanosomatids (Table I).^23^ GO analysis of the identified peptidases revealed a total of six biological processes: proteolysis involved in protein catabolism, ubiquitin-dependent protein catabolism, cell adhesion, protein deubiquitination, proteasome-mediated ubiquitin-dependent protein catabolism and protein processing involved in protein targeting to mitochondrion (Table II). A significant abundance of processes related to proteasome activity were identified, involving four biological process and 26 peptidases. In addition, a correlation was observed between the detected GP63/leishmanolysin peptidases and the cellular adhesion process.

**TABLE II:** Biological processes of *Strigomonas culicis* peptidases according to Gene Ontology (GO)

GO terms	Biological process	Occurrences	GenBank IDs
GO:0051603	proteolysis involved in protein catabolic process	11	EPY26142.1, EPY27567.1, EPY30848.1, EPY37086.1, EPY36921.1, EPY35086.1, EPY34301.1, EPY33242.1, EPY20654.1, EPY24218.1, EPY33248.1
GO:0006511	ubiquitin-dependent protein catabolic process	12	EPY18793.1, EPY19321.1, EPY19892.1, EPY20791.1, EPY25161.1, EPY26229.1, EPY20661.1, EPY27296.1, EPY27906.1, EPY31083.1, EPY25738.1, EPY33812.1,
GO:0007155	cell adhesion	7	EPY37270.1, EPY36381.1, EPY23056.1, EPY20885.1, EPY19626.1, EPY19508.1, EPY32300.1
GO:0016579	protein deubiquitination	6	EPY18793.1, EPY19321.1, EPY19892.1, EPY20791.1, EPY26229.1, EPY25161.1
GO:0043161	proteasome-mediated ubiquitin-dependent protein catabolic process	3	EPY26964.1, EPY37099.1, EPY27906.1
GO:0006627	protein processing involved in protein targeting to mitochondrion	2	EPY21346.1, EPY22990.1

**Fig. 2:**
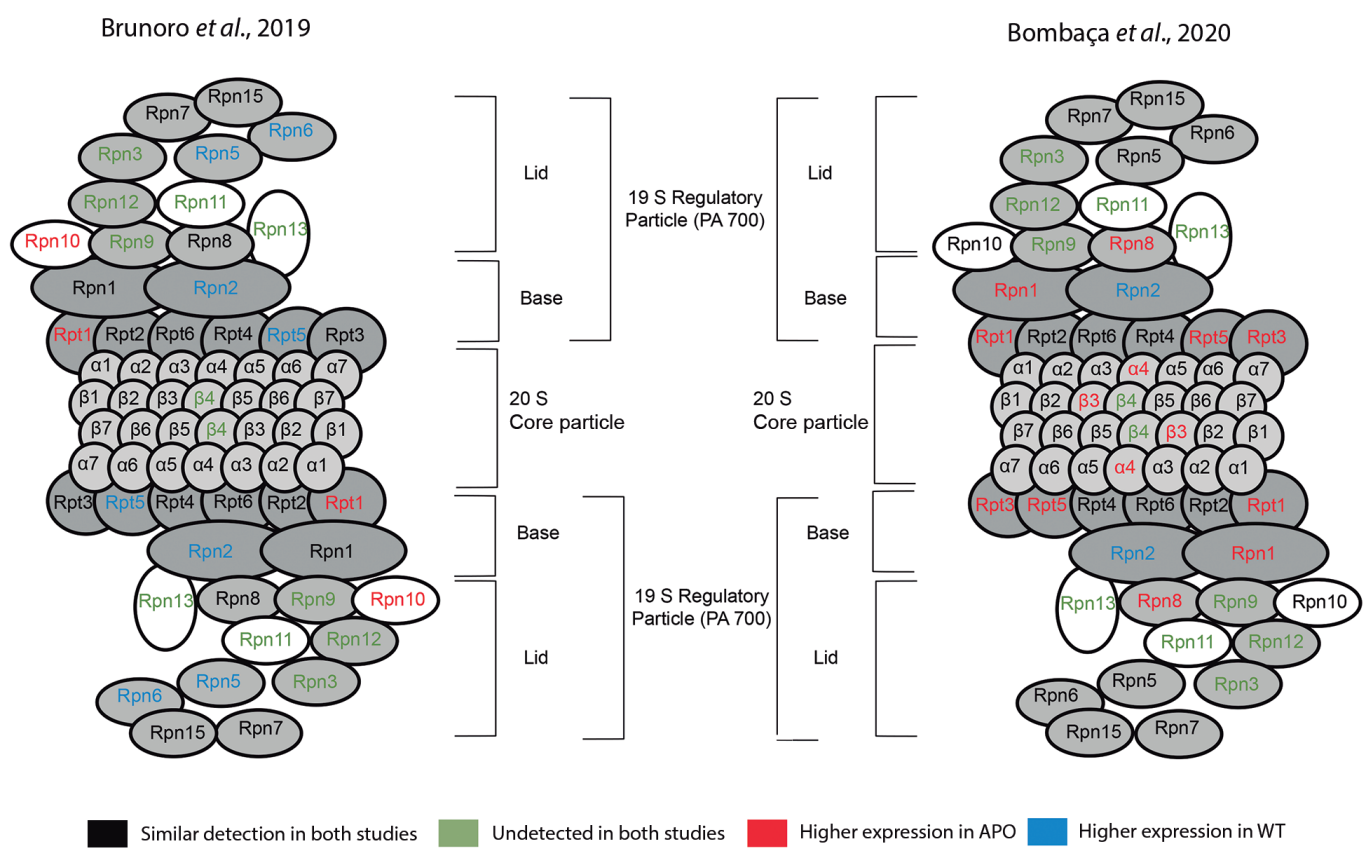
KEGG proteasome pathway scheme of the differential subunits’ expression between the wild-type (WT) and the aposymbiotic (APO) strains of *Strigomonas culicis*. Brunoro’s proteome^10^ on the left and in Bombaça’s proteome^11^ on the right. Proteasome subunits not identified in the *S. culicis* proteome are illustrated in green, identified proteins not modulated are represented in black, those up-modulated in the WT strain are shown in blue, and peptidases with a greater expression in the APO strain are represented in red.

The proteasome system comprises a key component of the ubiquitin-proteasome system (UPS) responsible for sustaining the cellular balance by selective degradation. This protein complex machinery comprises a regulatory 19S element associated with subunits like the regulatory particle ATPase (Rpt) and regulatory particle non-ATPase (Rpn), and a core 20S particle composed of alfa1-7 (α1-7) and beta1-7 (β1-7) subunits. Structurally like its mammalian counterpart, the trypanosomatid proteasome plays a vital role in protein turnover and degradation of ubiquitinated targets, with distinct catalytic activities in its β subunits.^24^ Given the abundance of proteasome-associated processes, it is intriguing to explore the proteasome pathway in KEGG. Our search in the proteome from Brunoro’s proteome^10^ reported an increased expression of Rpt1 and Rpn10 regulatory subunits in the APO strain, contrasting with an enhanced expression of Rpt5, Rpn2, Rpn5, and Rpn6 regulatory subunits in the WT strain. In Bombaça’s proteome^11^ an increased expression of seven subunits in the APO strain were detected, including the catalytic subunits α4 and β3, the regulatory subunits Rpt1, Rpt3, Rpt5, Rpn1, and Rpn8, but the Rpt8 regulatory subunit was enhanced in WT (Fig. 2). The β4 catalytic subunit and five regulatory subunits (Rpn3, 9, 11, 12, and 13) were not detected in both proteomes. The differential expression of proteasome subunits between strains suggests cellular adaptations in protein degradation by the dynamic interplay between symbiotic associations and cellular proteostasis. Each subunit plays specific roles in substrate recognition, unfolding, energy provision, and catalytic activity within the proteasome complex. For instance, Rpt1 and Rpt5 serve as ATPase subunits, providing energy for substrate unfolding and translocation, while Rpn10 and Rpn1 act as non-ATPase regulatory subunits crucial for substrate recognition and deubiquitination.^25^ Therefore, subunit expression differences may reflect variable cellular requirements for protein degradation, responses to environmental stressors, or adaptations to specific growth conditions between the strains. Unfortunately, it was not possible to compare the proteasome activity from the crude extracts of WT and APO strains employing the Z-LLL-AMC substrate (data not shown).

A total of 43 proteins (~ 48% of the total) showed changes in expression levels among strains, with metallo- and cysteine peptidases being the most identified across both proteomes [Supplementary data (Table III)]. Peptidases were categorised into two groups based on strain exclusivity and higher expression levels (Table III). In Brunoro’s proteome,^10^ 11 peptidases were exclusively detected in WT strain, 10 of them are cysteine peptidases and one is a threonine peptidase. In addition, a significantly higher expression level was detected in 1 metallo and 1 cysteine peptidases. In contrast, 20 peptidases were exclusively detected in the APO strain: comprising metallo (15), cysteine (4), and serine peptidases (1); and a higher expression of one metallopeptidase was reported. Similarly, in Bombaça’s proteome,^11^ only 1 cysteine peptidase was exclusively detected in the WT strain, while 1 metallo, two cysteine, and 1 threonine peptidases were exclusively detected in the APO strain. Additionally, six peptidases were highly expressed in the APO strain, including two metallo and three cysteine, in opposition of two metallopeptidases in WT (Table III). The eight metallopeptidases annotated as GP63/leishmanolysin and the 11 cysteine peptidases of the calpain family were the most modulated peptidases, pointing towards a direction for further analysis.

**TABLE III:** Differentially detected peptidases among *Strigomonas culicis* strains within the proteomes. Peptidases were classified in Brunoro’s proteome^10^ as exclusively detected in wild-type (WT) (●) or aposymbiotic (APO) (○) parasites, and highly expressed in WT (■) or APO (□). In Bombaça’s proteome,^11^ they were exclusively detected in WT (▲) or APO (∆), and highly expressed in WT (♦) or APO (◊)

Class	GenBank ID	Name	Group
Metallo	EPY27615.1	aminopeptidase	■
	EPY24850.1	mitochondrial processing peptidase	♦
	EPY27707.1	mitochondrial processing peptidase	
	EPY34173.1	metallo-peptidase	□
	EPY22990.1	mitochondrial intermediate peptidase	○
	EPY21346.1	metallo-peptidase	
	EPY20166.1	acetylornithine deacetylase	
	EPY19324.1	acetylornithine deacetylase	
	EPY26176.1	ATP-dependent zinc metallopeptidase	
	EPY37270.1	Leishmanolysin	
	EPY36381.1	Leishmanolysin	
	EPY23056.1	Leishmanolysin	
	EPY20885.1	Leishmanolysin	
	EPY19626.1	Leishmanolysin	
	EPY19508.1	Leishmanolysin	
	EPY32300.1	leishmanolysin-like	
	EPY21878.1	methionyl aminopeptidase	
	EPY23120.1	carboxypeptidase Taq	
	EPY21441.1	leucyl aminopeptidase	○,∆
	EPY23119.1	cytosol alanyl aminopeptidase	◊
	EPY23974.1	Aminopeptidase	
Cysteine	EPY31224.1	calpain-like cysteine peptidase	■, ◊
	EPY19819.1	calpain-like cysteine peptidase	○
	EPY28743.1	calpain-like cysteine peptidase	
	EPY18674.1	calpain-like cysteine peptidase	
	EPY23907.1	cysteine peptidase C	●
	EPY26229.1	cysteine peptidase	
	EPY30438.1	inhibitor of cysteine peptidase	
	EPY33460.1	calpain-like cysteine peptidase	
	EPY20021.1	calpain-like cysteine peptidase	
	EPY19174.1	calpain-like cysteine peptidase	
	EPY19892.1	ubiquitin carboxyl-terminal hydrolase 5/13	
	EPY19321.1	ubiquitin carboxyl-terminal hydrolase 5/13	
	EPY25227.1	lysosomal/endosomal membrane protein p67	
	EPY29273.1	calpain-like cysteine peptidase	▲
	EPY31483.1	cysteine peptidase	◊
	EPY18645.1	calpain-like cysteine peptidase	
	EPY18663.1	cysteine peptidase	∆
	EPY19437.1	calpain-like cysteine peptidase	●,∆
Threonine	EPY24218.1	ATP-dependent HslUV protease	●
	EPY26964.1	20S proteasome subunit beta 3	◊
	EPY25738.1	20S proteasome subunit alpha 4	∆
Serine	EPY34446.1	dipeptidase E	○

The exclusively detection of leucyl aminopeptidase EPY21441.1 in APO comprises another peptidase which deserves attention. There are few reports about this metallopeptidase in trypanosomatids. A study in *T. brucei* demonstrated that its orthologous TbLAP1 is a mitochondrial protein associated with kDNA during the cell cycle.^26^ This protein localises to kDNA and the proteinaceous link connecting progeny kDNAs at late stages of segregation, a structure named as nabelschnur. Therefore, it´s assumed that TbLAP1 could participate at the kDNA replication. In *S. culicis*, the kinetoplast segregation happen close to karyokinesis,^27^ as a late event of cell cycle, in contrast with *T. brucei* which kinetoplast S phase initiates before the nuclear DNA synthesis. A major leucyl aminopeptidolytic activity was identified in all *T. cruzi* forms. However, since biosynthetic pathways for essential amino acids, including leucine, are lacking in *T. cruzi*, this leucyl aminopeptidase was assumed to have a function in nutritional supply.^28^

**Fig. 3:**
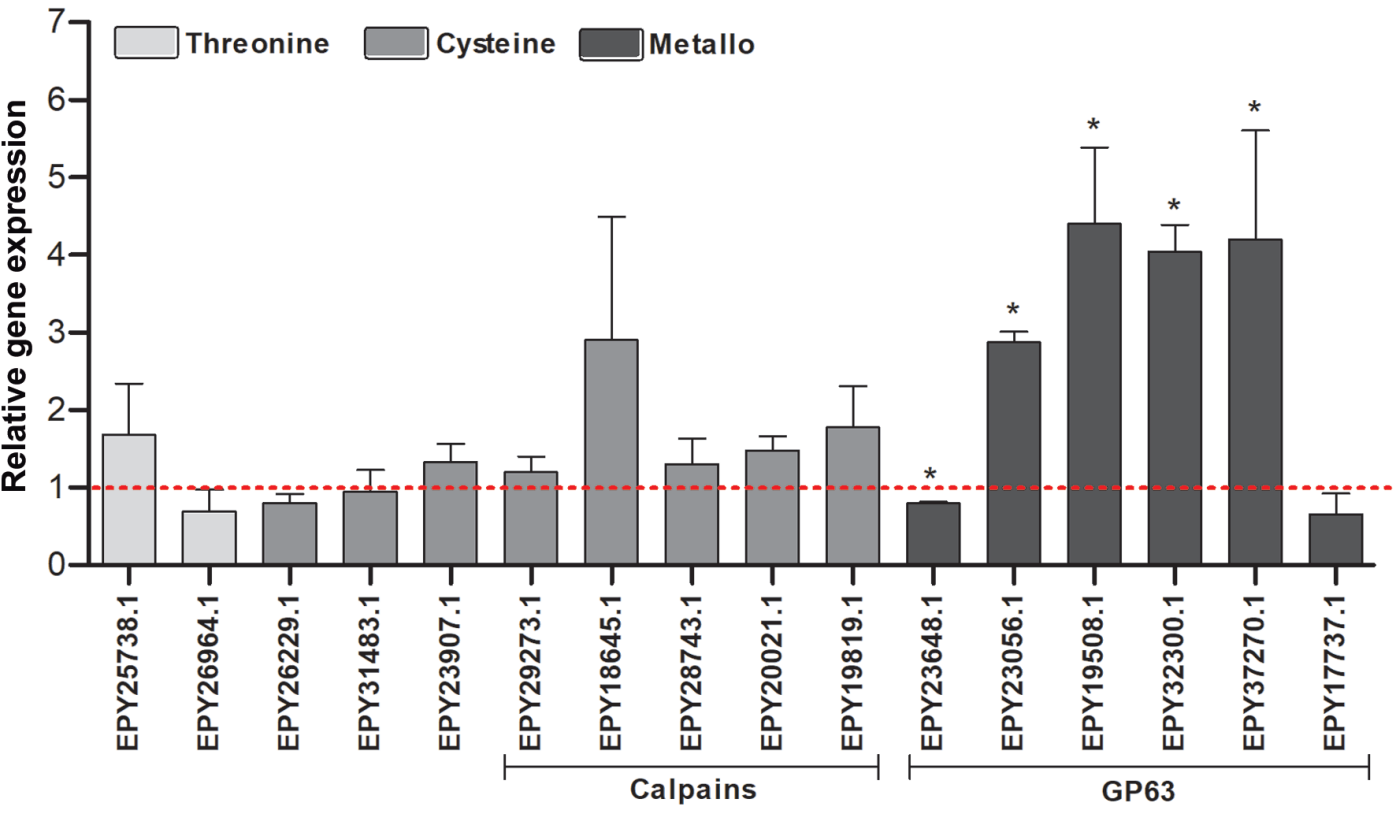
relative expression of the wild-type (WT) and the aposymbiotic (APO) strains of *Strigomonas culicis* by real-time reverse transcriptase quantitative polymerase chain reaction (RT-qPCR). The dashed red line represents the gene expression of the WT strain. The bars correspond to the ratio of the difference between the APO strain and the WT strain. Light grey bars represent threonine peptidases, dark grey bars represent cysteine peptidases, and black bars represent metallopeptidases. Statistical tests performed: Student’s t-test and Mann-Whitney test. *p < 0.05.

*Differential expression of GP63 genes between WT and APO strains* - Considering the differential expression observed in the proteomic studies, specific peptidases were selected for further validation by gene expression analysis. From the 36 sequences identified as differentially modulated between WT and APO strains, 16 were selected based on the presence of a catalytic domain and the absence of an identical copy between each other. In summary, two sequences were threonine peptidases (α4 and β3 subunits of the 20s proteome), eight were cysteine peptidases (1 cysteine peptidase C, two cysteine peptidases, and five calpains), and six were metallopeptidases (GP63 sequences). In addition, the paraflagellar rod (PFR) and actin (ACT) genes were used as endogenous control. The RT-qPCR analysis revealed significant expression differences in five GP63 sequences: four were highly expressed in the APO strain, whereas 1 was enhanced in WT (Fig. 3). Just 1 GP63 sequence did not reported significant expression between the strains.

The up-regulation of GP63 in the APO strain is intriguing due to its key roles in promastigotes from the *Leishmania* genus. GP63 is a zinc-dependent metallopeptidase implicated in invasion, survival, and virulence during the interaction with the vertebrate and invertebrate hosts, as extensively studied.^29^ ^,^ ^30^ The up-regulation of four of the six GP63 in the non-virulent APO strain raises questions about its roles modulating virulence and host interactions. Further investigations are needed to understand the dynamics of GP63 in *S. culicis* biology, including if this increased expression could be compensatory or reflective of alterations in host-parasite interactions.

On the other hand, eight of the analysed cysteine peptidases showed no significant differences in gene expression between *S. culicis* strains. However, it is intriguing to explore the abundance of calpains in our analysis. Calpains are calcium-dependent cysteine proteases responsible for a broad range of cellular processes, such as signal transduction, cytoskeletal remodelling, and apoptosis.^21^ Trypanosomatids harbour a large and diverse family of calpain sequences in their genomes, but these peptidases are considered to play a structural, non-proteolytic function.^19^ The lack of a significant modulation of calpains detected by RT-qPCR analysis contrasts with the proteomic data, but still open some questions about the functions played by calpains in *S. culicis*. Nevertheless, trypanosomatid’s gene expression control essentially occurs at the post-transcriptional level since constitutive polycistronic transcription of protein-coding genes and *trans*-splicing are usual in trypanosomatids. However, it has been shown a link between differential expression levels of calpains’ transcripts and protein expression in *T. brucei, T. cruzi* and *L. braziliensis*.^13^ ^,^ ^31^ ^,^ ^32^

Threonine peptidases are vital enzymes for protein degradation and cellular regulation, in which the proteasome is the most extensively studied enzymatic complex. The α4 subunit is associated with the ubiquitin-dependent protein catabolic process (GO:0006511), orchestrating the breakdown of proteins or peptides through hydrolysis of peptide bonds. Conversely, the β3 subunit is linked to the proteasome-mediated ubiquitin-dependent protein catabolic process (GO:0043161), which shares the same fundamental mechanism of protein breakdown initiated by ubiquitin attachment and mediated by the proteasome.^25^ Although the proteomic data from previous studies reported a higher expression of α4 and β3 subunits in APO parasites, our RT-qPCR analysis did not exhibit significant differences between *S. culicis* strains.

**Fig. 4:**
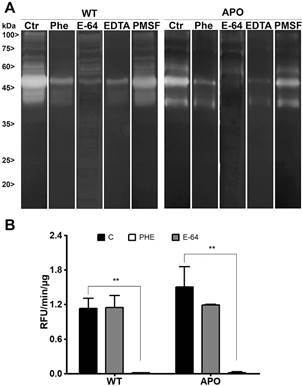
proteolytic activity of *Strigomonas culicis* wild-type (WT) and aposymbiotic (APO) crude extracts by Gelatine-SDS-PAGE and in-solution Gelatine-FITC. (A) The gels were incubated in 50 mM phosphate buffer pH 5.5 for 72 h at 37ºC. Zymograms were incubated in 1 mM DTT with or without (control) the following inhibitors: 1 mM PMSF, 10 mM EDTA, 10 μM E-64, and 10 mM 1,10-phenanthroline (Phe/PHE). The numbers on the right indicate molecular weight markers. (B) The in-solution activity was assessed by measuring the hydrolysis of the fluorogenic substrate Gelatine-FITC (100 mM) at 37ºC for 40 min. WT and APO proteolysis were measured in 100 mM glycine‐NaOH pH 10.0 buffer in the presence or absence of 10 μM 1,10-phenantroline and 10 μM E-64. Results represent the mean ± standard deviation of three independent experiments performed in triplicate, and statistical analysis was performed using 2-way ANOVA.

*Protease expression pattern of peptidases in both strains* - SDS-PAGE gels are commonly used to access peptidase activity in cellular systems, including protozoa, offering a valuable tool to analyse their prevalence and expression.^33^ Here, zymography with gelatine and casein substrates was employed to evaluate enzymatic activity in *S. culicis* WT and APO strains*.* Our results at pH 5.5 presented similar degradation profiles as observed previously by our research group, whereas the proteolytic activity could be observed in six bands around 76, 64, 55, 50, 40 and 35 kDa^33^ (Fig. 4A). No additional band was observed in casein substrate or in gels incubated in pH 7.4 (data not shown). Additionally, the lower bands (35 to 50 kDa) were inhibited by cysteine peptidase inhibitor E-64, while the metallopeptidase inhibitory PHE restrained the upper bands (55 to 76 kDa). The serine peptidase inhibitor PMSF had no significant effect, probably due to (i) the minor presence of this class of peptidase in trypanosomatid genomes;^23^ or (ii) the absence of proteolysis in the incubation conditions employed, such as time, temperature, ions, ionic strength and substrates. Employing gelatine as soluble substrate, the proteolytic activity of both extracts was significantly suppressed in the presence of PHE, while E-64 inhibition was not significant, suggesting a predominant enzymatic activity from metallopeptidases employing the manufacturers’ pH 7.6 Tris-HCl buffer (Fig. 4B). No activity was archived using alternative buffers (data not shown).

**Fig. 5:**
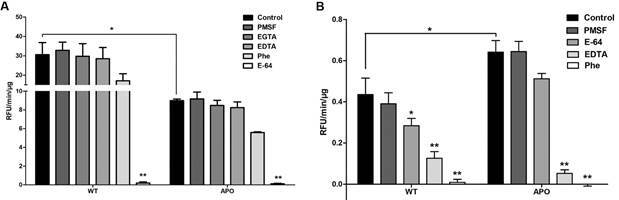
proteolytic activity of *Strigomonas culicis* wild-type (WT) and aposymbiotic (APO) crude extracts by in-solution assays. Cysteine peptidase activity was assessed by monitoring the hydrolysis of fluorogenic substrate z-Phe-Arg-AMC (100 mM) at 37ºC for 30 min in 100 mM sodium phosphate buffer, pH 5.5 (A). GP63 activity was evaluated using the fluorogenic substrate MMP13 (100 mM) at 37ºC for 1 h in 0.1 M glycine-NaOH, 1 mM CaCl_2_ buffer, pH 10.0 (B). Both assays were conducted in the presence or absence of 10 μM 1,10-phenantroline and 10 μM E-64. Results represent the mean ± standard deviation of three independent experiments performed in triplicate, and statistical analysis was performed using 2-way ANOVA.

The cysteine-specific substrate Z-Phe-Arg-AMC exhibited significantly higher activity in the WT strain than in APO, notably inhibited by E-64, thus confirming cysteine-type activity (Fig. 5A). These findings support proteomic observations, which consistently showed a higher detection of cysteine peptidases in WT compared to the APO strain. The increased degradation reported by the WT strain corroborates findings from heteroxenic trypanosomatids, since these enzymes seems to play pivotal roles in drug exposure,^34^ ^,^ ^35^ disclose essential surface receptors during parasite interaction,^15^ and enable the adhesion to the insect midgut.^36^ Unfortunately, attributing these results to calpains was unfeasible since although massive efforts have been made to identify the activity of trypanosomatids’ calpains, data from *T. brucei* and *T. cruzi* studies suggest that these proteins should play structural, non-proteolytic functions.^19^ However, it is important to note that other cysteine peptidases are the main active molecules in trypanosomatids, such as homologous of cruzipain from *T. cruzi* detected in previous studies,^37^ or even the cysteine peptidase C (EPY23907.1) reported as higher expressed in Brunoro’s proteome.^10^ In *T. cruzi* epimastigotes, cruzipain is found in reservosomes, a lysosome-like organelle where protein degradation occurs for nutritional purposes and is also crucial for the interaction of the parasite with the invertebrate host.^37^ No homologous of cruzipain or cathepsin L were identified in both proteomic data analysed.

The matrix metallopeptidase fluorogenic substrate MMP13 was used to assess the GP63 activity from *S. culicis* strains (Fig. 5B). A significant higher proteolytic activity could be observed in the APO strain compared to the WT strain. The almost completely inhibition by PHE and partially inhibition by the ion chelator EDTA corroborate the metallopeptidase activity. Metabolic shifts triggered by the absence of some molecules, or even some pathways, could be regulating GP63 expression. For instance, GP63 was reported to be largely expanded in the genome of the lizard parasite *L. tarentolae* but exhibits lower proteolytic activity than human pathogenic *Leishmania* spp., which might be attributable to the less negative electrostatic potential observed in the molecular dynamic simulations.^12^ Accordingly, *T. cruzi* GP63 are extensively expanded with more than 420 predicted genes and pseudogenes, but the enzymatic activity of *T. cruzi* extracts is rather weak, only obtained in the presence of proteolytic inhibitors from the other peptidase classes.^38^

**Fig. 6:**
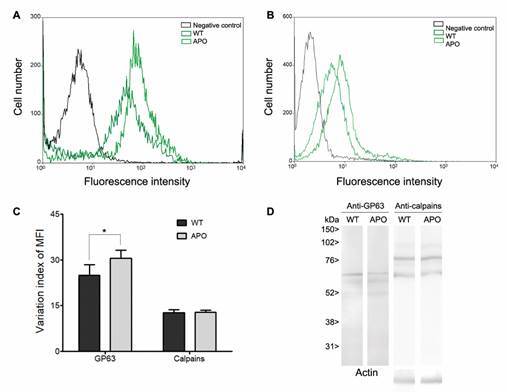
protein expression pattern of calpains and GP63 recognized by the anti-tritryp-calpain and anti-GP63 antibodies in *Strigomonas culicis* wild-type (WT) and aposymbiotic (APO) strains. Panels A and B demonstrates flow cytometry analysis of WT (dark green) and APO (light green) parasites labelled with anti-tritryp-calpain or anti-GP63 antibodies, respectively, alongside autofluorescence control (black). Representative data from one experiment, analysing 10,000 cells, reflected results from three independent experiments. Panel C displays the variation index of the mean fluorescent intensity (MFI) from each strain, obtained by dividing the MFI from labelled parasites by the autofluorescence controls from the flow cytometric analysis. Standard deviation of the mean was indicated by bars, with * indicating statistically significant differences between strains MFI (p < 0.05). Panel D presents immunoblotting results of total cellular extracts from the mid-log phase of the two *S. culicis* strains, probed with anti-calpain and anti-GP63 antibodies. SDS-PAGE protein standards were provided for reference, and an anti-actin antibody served as a loading control.

*GP63 and calpains at a protein level* - Finally, the protein expression profile of *S. culicis* GP63 and calpains were compared by western blotting and flow cytometry. At first, our results revealed that the binding of anti-GP63 antibodies was significantly higher in permeabilised parasites, which indicates that *S. culicis* GP63 are located mainly in intracellular compartments, although low levels were detected on the cell surface (data not shown). The membrane labelling is a prominent issue, since there is substantial data suggesting that GP63 homologues found in the surface of monoxenic trypanosomatids play crucial roles in the parasite uptake of essential components through degradation of gut content, as well as in the binding to the insect epithelial cells.^39^ Notwithstanding, the flow cytometric analysis provided measurements for a comparative expression of WT and APO strains through the variation index of the mean of fluorescence intensity (MFI). In this sense, the variation index of MFI from permeabilised parasites a higher expression of GP63 in APO parasites in comparison with the WT strain (Fig. 6A, C). This data agrees with the proteomic findings, and with our gene expression and enzymatic results. However, in other endosymbiont-harbouring trypanosomatid, *Angomonas deanei*, a 2-fold increased expression of GP63 was reported in WT parasites by flow cytometry.^40^

Aiming to evaluate the global shifts in CysPc-containing proteins in *S. culicis* WT and APO strains, a previously validated polyclonal antibody targeting a consensus polypeptide sequence from trypanosomatids’ calpains (LEKAYAKLHGSY) was employed.^13^ ^,^ ^32^ No statistically significant difference could be detected by the anti-tritryp-calpain antibody in permeabilised parasites from both strains, corroborating our gene expression analysis (Fig. 6B, C). Although trypanosomatids calpains have been extensively evaluated by flow cytometric analysis, until nowadays there is no report regarding the global calpain abundance among *S. culicis* strains. In *A. deanei*, an inconclusive expression profile was reported, whereas an increased labelling was detected by an antibody raised against *Drosophila melanogaster* calpain-like molecules in the endosymbiont-harbouring strain, but the opposite was observed employing the monoclonal anti-CAP5.5 raised against the cytoskeleton-associated calpain from *T. brucei*.^41^ Moreover, calpains exhibited consistent expression profile between *L. braziliensis* recently isolated and culture-adapted parasites, whereas the maintenance of *T. cruzi* in the axenic culture for a long time led to a decreased expression.^13^ ^,^ ^32^ A better understanding of the expression of calpains in trypanosomatids is still crucial to explain the roles displayed by these molecules.

In addition to flow cytometric analysis, western blotting assays were performed with cellular extracts of *S. culicis* WT and APO parasites employing the same antibodies. As a result, it was possible do identify three bands migrating near the 63 kDa after the cross-reaction with anti-GP63 antibodies (Fig. 6D). No statistically significant difference in the intensity of reactive bands was detected between WT and APO strains, as measured by densitometric analysis (data not shown). The reactive bands migrate at approximately 60, 56 and 50 kDa, which are a remarkably similar molecular mass of leishmanolysin-like genes EPY37270.1, EPY20885.1 and EPY23056.1 previously reported in proteomic data. Although a direct association between the predicted molecular mass with those detected experimentally was not unequivocal, it is important to note that leishmanolysin undergoes post-transcriptional changes that could modify the molecular mass in the western blotting.^42^ Additionally, the anti-tritryp-calpain antibody strongly recognised in *S. culicis* extracts two polypeptides migrating at approximately 78 and 58 kDa, and two faint bands at 100 and 70 kDa (Fig. 6D). This complex expression profile was expected since the antibody was raised against a consensus polypeptide from trypanosomatids’ calpains, and related results were reported in *L. braziliensis* and *T. cruzi* extracts.^13^ ^,^ ^31^ Accordingly, 11 different calpains in WT and APO strains were identified in the proteomic studies, which could make feasible the detection of calpains with predicted molecular masses ranging from 53 to 278 kDa. Notwithstanding, calpains suffer an autolytic conversion in the presence of calcium,^21^ and trypanosomatid calpain orthologues may undergo post-transcriptional modifications.^43^

In conclusion, our analyses shed light on the endosymbiont’s role in modulating GP63 and cysteine peptidases expression, suggesting its involvement in crucial cellular processes for the parasite. The results reported here on GP63 expression and activity could inspire further investigation into the relationship between *S. culicis* and its endosymbiont. Altogether, these findings underscore a potential impact of symbiosis, which could provide insights about *S. culicis* adaptive strategies and contribute to a better understanding of its biology.
