# Supplementary material for: Differential expression of peptidases in Strigomonas culicis wild-type and aposymbiotic strains: from proteomic data to proteolytic activity
Source: Mem Inst Oswaldo Cruz. 2024 Dec 9;119:e240110. doi: 10.1590/0074-02760240110 (PMC11654740; doi:10.1590/0074-02760240110)
Supplement: Supplementary file 1 [file 1678-8060-mioc-119-e240110-s.pdf]

TABLE I

Oligonucleotides sequences used in real-time reverse transcriptase quantitative polymerase chain reaction (RT-qPCR) assays

GenBank ID	Gene name		Oligonucleotide sequences (5'-3')	Product size
EPY25738.1	20S proteasome subunit alpha 4	Fw	CATCGACCTCCTCGTGCGCT	112 bp
		Rv	GCCGTTCTCGTTGAAGCCGC	
EPY26964.1	20S proteasome subunit beta 3	Fw	GGAACCTCCGCTGTACTGG	107 bp
		Rv	CCTGTTGACGATGTTAGTATCTGATAT	
EPY31483.1	cysteine peptidase	Fw	CCGCGCATCTCAGAAGGC	80 bp
		Rv	TGGGCATGCCTCACTCTCT	
EPY23907.1	cysteine peptidase C	Fw	CGAGCAGAGCTACGGCGTCA	136 bp
		Rv	GCCGGTGACGTGCTGGTAGA	
EPY26229.1	cysteine peptidase	Fw	CTCTGTGATCAGCATGGGCA	91 bp
		Rv	AATGTAATCCACCGCTCGCT	
EPY29273.1	calpain-like cysteine peptidase	Fw	GGACGACGAGGTACGGGGAG	102 bp
		Rv	TCGACAACGACCACCCGACG	
EPY18645.1	calpain-like cysteine peptidase	Fw	CCCGTGCGACAGCAACAACC	129 bp
		Rv	TGCGGCGTGCAGTTCTCCAT	
EPY28743.1	calpain-like cysteine peptidase	Fw	TGTGTGTTCTCGAGTGCCTC	96 bp
		Rv	AACCTGGGCCTTCTACAACG	
EPY20021.1	calpain-like cysteine peptidase	Fw	GGAGGATGTTGCCACGGATT	105 bp
		Rv	TCATACACACCGAACTCGCG	
EPY19819.1	calpain-like cysteine peptidase	Fw	TGACCTCCGCAATGTAGTCG	130 bp
		Rv	GCCTTCTACAACGACAGCCT	
EPY23648.1	leishmanolysin	Fw	GGATGCCATAGAGGAGCGTC	96 bp
		Rv	AAGGTCAGGTGCTTCTTCGG	
EPY23056.1	leishmanolysin	Fw	GTCGGTAATATCGCGCCACT	98 bp
		Rv	CACCCACGTAGAGCAGGAAG	
EPY19508.1	leishmanolysin	Fw	TCGGACAGCTGGATCGATTG	252 bp
		Rv	CCCTTAAGCAACAAAAATAATGTCAA	
EPY32300.1	leishmanolysin	Fw	GCTGTTCTCGCGACTTCCAA	80 bp
		Rv	TCCAGCTCCATGAAGGACTG	
EPY37270.1	leishmanolysin	Fw	TGCCATGTCTTCTTTTGCATTTCT	104 bp
		Rv	AGCTACTATTATTCCCTTAAGCAACA	
EPY17737.1	leishmanolysin-like	Fw	ACAGTAGTGTAGGGGAGCGT	141 bp
		Rv	GGCAGAACTTCTCCGTGTCA	
EPY34789.1	paraflagellar rod	Fw	CGGGAGAACGTGGAGCGACA	111 bp
		Rv	TTCGATGCGGCGCTTGACCT	
EPY37147.1	Actin Bombaça et al.[11]	Fw	TGCCATTCAACTGTCGTCCT	76 bp
		Rv	GTATGGGTTTCGCCGTCCA	

Fw: foward primer; Rv: reverse primer; bp: base pair.

CC BY

TABLE II

Strigomonas culicis peptidase sequences retrieved from GenBank

GenBank ID	Name	MEROPS ID	Family / Subfamily	Enzymatic class	Domains	Predict molecular mass (kDa)
EPY32210.1	mitochondrial processing peptidase			Metallopeptidase	Peptidase M16 e Peptidase M16 C (Pfam)	54,17
EPY27707.1	mitochondrial processing peptidase			Metallopeptidase	Peptidase M16 (Pfam)	50,46
EPY24850.1	mitochondrial processing peptidase	MER0922125	M16B	Metallopeptidase	Peptidase M16 e Peptidase M16 C (Pfam)	54,17
EPY23344.1	mitochondrial processing peptidase	MER0898762	M16A	Metallopeptidase	Peptidase M16 e Peptidase M16 C (Pfam)	55,93
EPY35284.1	mitochondrial processing peptidase			Metallopeptidase	Peptidase M16 (Pfam)	41,48
EPY29207.1	mitochondrial processing peptidase			Metallopeptidase	Peptidase M11	36
EPY34199.1	mitochondrial processing peptidase			Metallopeptidase	Peptidase M16 C (Pfam)	56,99
EPY28378.1	mitochondrial processing peptidase			Metallopeptidase	Peptidase M16 e Peptidase M16 C (Pfam)	49,73
EPY24713.1	mitochondrial processing peptidase	MER0928259	M16B	Metallopeptidase	Peptidase M16 e Peptidase M16 C (Pfam)	49,73
EPY27820.1	mitochondrial processing peptidase			Metallopeptidase	Peptidase M16 C (Pfam)	49,11
EPY33640.1	mitochondrial processing peptidase			Metallopeptidase	Peptidase M11	40
EPY34308.1	signal peptidase protein			Serine peptidase	Peptidase S24 (Pfam)	24,32
EPY31660.1	signal peptidase protein			Serine peptidase	Peptidase S24 (Pfam)	21,86
EPY31133.1	signal peptidase protein			Serine peptidase	Peptidase S24 (Pfam)	19,74
EPY30613.1	signal peptidase protein	MER1052675	S26B	Serine peptidase	Peptidase S24 (Pfam)	24,32
EPY25514.1	mitochondrial inner membrane signal peptidase	MER1044355	S26A	Serine peptidase	Peptidase S24 (Pfam)	22,67
EPY36098.1	cysteine peptidase C			Cysteine peptidase	Pept_C1 (SMART - Interpro)	22,4
EPY23907.1	cysteine peptidase C	MER0704373	C1A	Cysteine peptidase	Pept_C1 (SMART - Interpro)	23,4
EPY26229.1	cysteine peptidase			Cysteine peptidase	ZnF_UBP e UBA (SMART - Interpro)	79,58
EPY25815.1	cysteine peptidase			Cysteine peptidase	UCH (Pfam)	95,72
EPY34688.1	cysteine peptidase	MER0707153	C2A	Cysteine peptidase	DUF1935 (Pfam) e CysPc (SMART - Interpro)	87,03
EPY31483.1	cysteine peptidase	MER0706650	C2A	Cysteine peptidase	DUF1935 (Pfam) e CysPc (SMART - Interpro)	87,03
EPY26882.1	cysteine peptidase					58,66
EPY23049.1	cysteine peptidase	MER0705939	C2A	Cysteine peptidase	CysPc (SMART - Interpro)	67,84
EPY18663.1	cysteine peptidase			Cysteine peptidase	2xRPT1 (Prospero)	359,42
EPY34761.1	peptidase M20/M25/M40	MER0851768	M20F	Metallopeptidase	Peptidase M20 Dimer (Pfam)	30,41
EPY20780.1	peptidase M20/M25/M41			Metallopeptidase	Peptidase M20 Dimer (Pfam)	32,34
EPY33312.1	peptidase M20/M25/M42			Metallopeptidase	Peptidase M20 Dimer (Pfam)	52,02
EPY29080.1	peptidase M20/M25/M43			Metallopeptidase	Peptidase M20 Dimer (Pfam)	52,02
EPY28238.1	peptidase M20/M25/M44			Metallopeptidase	Peptidase M20 Dimer (Pfam)	52,02
EPY27770.1	peptidase M20/M25/M45			Metallopeptidase	Peptidase M20 Dimer (Pfam)	52,02
EPY32795.1	inhibitor of cysteine peptidase			Cysteine peptidase	Inhibitor I42 Domain	11,82
EPY30438.1	inhibitor of cysteine peptidase			Cysteine peptidase	Inhibitor I42 Domain	12,82
EPY23888.1	peptidase T	MER0932050	M20B	Metallopeptidase	Peptidase M28 (Pfam)	46,09
EPY22990.1	mitochondrial intermediate peptidase	MER0818143	M3A	Metallopeptidase	Peptidase M3 (Pfam)	77,55
EPY19905.1	mitochondrial intermediate peptidase		M3A	Metallopeptidase	Peptidase M3 (Pfam)	78,55
EPY22055.1	cysteine peptidase A			Cysteine peptidase	Inhibitor I29 e Pept_C1 (SMART - Interpro), e DUFF 3586 (Pfam)	58,98
EPY21129.1	cysteine peptidase A	MER0702575	C1, C1.076	Cysteine peptidase	Inhibitor I29 e Pept_C1 (SMART - Interpro), e DUFF 3586 (Pfam)	72,39
EPY37014.1	Bem46-like serine peptidase			Serine peptidase	Abhydrolase 6 (Pfam)	44,21
EPY25984.1	Bem46-like serine peptidase			Serine peptidase	Abhydrolase 6 (Pfam)	45,21
EPY35565.1	ATP-dependent HslUV protease			Threonine peptidase	Proteasome (Pfam)	14,58
EPY33248.1	ATP-dependent HslUV protease	MER1089424	T1B	Threonine peptidase	Proteasome (Pfam)	23,01
EPY31308.1	ATP-dependent HslUV protease	MER1094230	T1B	Threonine peptidase	Proteasome (Pfam)	16,45
EPY24218.1	ATP-dependent HslUV protease	MER1091871	T1B	Threonine peptidase	Proteasome (Pfam)	18,11
EPY33780.1	prenyl protein peptidase			Metallopeptidase	Abi (Pfam)	33,08
EPY31107.1	prenyl protein peptidase	MER0937074	M79	Metallopeptidase	Abi (Pfam)	33,08
EPY24470.1	prenyl protein peptidase			Metallopeptidase	Abi (Pfam)	24,57
EPY25905.1	serine peptidase			Serine peptidase	Abhydrolase 6 (Pfam)	70,77
EPY29672.1	serine peptidase			Serine peptidase	Hydrolase 4 (Pfam)	37,91
EPY23061.1	serine peptidase			Serine peptidase	Hydrolase 4 (Pfam)	37,91
EPY28464.1	serine peptidase			Serine peptidase	Rhomboid (Pfam)	44,17
EPY21398.1	serine peptidase			Serine peptidase	Rhomboid (Pfam)	38,04
EPY36883.1	calpain-like cysteine peptidase			Cysteine peptidase	DUF1935 (Pfam)	16,43
EPY34319.1	calpain-like cysteine peptidase			Cysteine peptidase	Fragmented CysPc (Smart - Interpro)	22,95
EPY34294.1	calpain-like cysteine peptidase			Cysteine peptidase	DUF1935 (Pfam)	19,33
EPY33831.1	calpain-like cysteine peptidase			Cysteine peptidase	DUF1935 (Pfam)	13,84
EPY33460.1	calpain-like cysteine peptidase			Cysteine peptidase	DUF1935 (Pfam)	13,97

GenBank ID	Name	MEROPS ID	Family / Subfamily	Enzymatic class	Domains	Predict molecular mass (kDa)
EPY32278.1	calpain-like cysteine peptidase	MER0707202	C2A	Cysteine peptidase	DUF1935 (Pfam) e CysPc (SMART - Interpro)	70
EPY31260.1	calpain-like cysteine peptidase			Cysteine peptidase	DUF1935 (Pfam) e CysPc (SMART - Interpro)	96,96
EPY31224.1	calpain-like cysteine peptidase			Cysteine peptidase	DUF1935 (Pfam)	17,42
EPY31079.1	calpain-like cysteine peptidase			Cysteine peptidase	DUF1935 (Pfam)	12,88
EPY31015.1	calpain-like cysteine peptidase			Cysteine peptidase	DUF1935 (Pfam) e CysPc (SMART - Interpro)	89,29
EPY30853.1	calpain-like cysteine peptidase			Cysteine peptidase	DUF1935 (Pfam) e CysPc (SMART - Interpro)	93,29
EPY30436.1	calpain-like cysteine peptidase	MER0706700	C2A	Cysteine peptidase	DUF1935 (Pfam) e CysPc (SMART - Interpro)	53,88
EPY30171.1	calpain-like cysteine peptidase			Cysteine peptidase	Fragmented CysPc (SMART - Interpro) e Calpain III (Pfam)	25,87
EPY29273.1	calpain-like cysteine peptidase	MER0706231	C2A	Cysteine peptidase	DUF1935 (Pfam) e CysPc (SMART - Interpro)	77,32
EPY29211.1	calpain-like cysteine peptidase	MER0706741	C2A	Cysteine peptidase	DUF1935 (Pfam) e CysPc (SMART - Interpro)	62,34
EPY29193.1	calpain-like cysteine peptidase			Cysteine peptidase	DUF1935 (Pfam) e CysPc (SMART - Interpro)	96,96
EPY29106.1	calpain-like cysteine peptidase			Cysteine peptidase		30,84
EPY28743.1	calpain-like cysteine peptidase			Cysteine peptidase	DUF1935 (Pfam) e CysPc (SMART - Interpro)	69,45
EPY28675.1	calpain-like cysteine peptidase	MER0706847	C2A	Cysteine peptidase	DUF1935 (Pfam) e CysPc (SMART - Interpro)	75,8
EPY27651.1	calpain-like cysteine peptidase	MER0707280	C2A	Cysteine peptidase	DUF1935 (Pfam) e CysPc (SMART - Interpro)	96,96
EPY26838.1	calpain-like cysteine peptidase	MER0706039	C2A	Cysteine peptidase	CysPc e Calpain_III (SMART - Interpro)	65,12
EPY26815.1	calpain-like cysteine peptidase	MER0706040	C2A	Cysteine peptidase	DUF1935 (Pfam) e CysPc (SMART - Interpro)	89,27
EPY25494.1	calpain-like cysteine peptidase			Cysteine peptidase	DUF1935 (Pfam) e CysPc (SMART - Interpro)	92,89
EPY24361.1	calpain-like cysteine peptidase			Cysteine peptidase	DUF1935 (Pfam) e CysPc (SMART - Interpro)	80,1
EPY24019.1	calpain-like cysteine peptidase	MER0707009	C2A	Cysteine peptidase	CysPc (SMART - Interpro)	43,83
EPY23567.1	calpain-like cysteine peptidase			Cysteine peptidase	DUF1935 (Pfam)	14,73
EPY22712.1	calpain-like cysteine peptidase	MER0706095	C2A	Cysteine peptidase	DUF1935 (Pfam) e CysPc (SMART - Interpro)	54,72
EPY22267.1	calpain-like cysteine peptidase			Cysteine peptidase	DUF1935 (Pfam) e CysPc (SMART - Interpro)	60,03
EPY20933.1	calpain-like cysteine peptidase	MER0705973	C2A	Cysteine peptidase	DUF1935 (Pfam) e CysPc (SMART - Interpro)	61,03
EPY20021.1	calpain-like cysteine peptidase	MER0707061	C2A	Cysteine peptidase	DUF1935 (Pfam) e CysPc (SMART - Interpro)	89,29
EPY19819.1	calpain-like cysteine peptidase	MER0706686	C2A	Cysteine peptidase	DUF1935 (Pfam) e CysPc (SMART - Interpro)	93,29
EPY19437.1	calpain-like cysteine peptidase	MER0707143	C2A	Cysteine peptidase	RPT1 (Propero) e CysPc (SMART - Interpro)	278,01
EPY19249.1	calpain-like cysteine peptidase	MER0706313	C2A	Cysteine peptidase	DUF1935 (Pfam) e CysPc (SMART - Interpro)	80,1
EPY19174.1	calpain-like cysteine peptidase			Cysteine peptidase	2xRPT2 e RPT3 (Prospero)	118,66
EPY18788.1	calpain-like cysteine peptidase			Cysteine peptidase	DUF1935 (Pfam) e CysPc (SMART - Interpro)	53,32
EPY18706.1	calpain-like cysteine peptidase	MER0706978	C2A	Cysteine peptidase	DUF1935 (Pfam) e CysPc (SMART - Interpro)	92,89
EPY18674.1	calpain-like cysteine peptidase			Cysteine peptidase	2xRPT1 (Prospero)	283,23
EPY18665.1	calpain-like cysteine peptidase			Cysteine peptidase	RPT4 e RPT3 (Prospero)	203,68
EPY18645.1	calpain-like cysteine peptidase	MER0706922	C2A	Cysteine peptidase	RPT1 (Propero) e CysPc (SMART - Interpro)	100,76
EPY17873.1	calpain-like cysteine peptidase			Cysteine peptidase	2xRPT1 (Prospero)	25,49
EPY17229.1	calpain-like cysteine peptidase			Cysteine peptidase	2xRPT1 (Prospero)	72,63
EPY17169.1	calpain-like cysteine peptidase	MER0706217	C2A	Cysteine peptidase	DUF1935 (Pfam) e CysPc (SMART - Interpro)	43,76
EPY16112.1	calpain-like cysteine peptidase	MER0705949	C2A	Cysteine peptidase	CysPc (SMART - Interpro)	74,26
EPY29320.1	insulysin	MER0919867	M16A	Metallopeptidase	Peptidase M16, M16 C e M16 M (Pfam)	118.88
EPY26831.1	insulysin			Metallopeptidase	Peptidase M16, M16 C e M16 M (Pfam)	114,45
EPY22943.1	insulysin	MER0714354	M16A	Metallopeptidase	Peptidase M16, M16 C e M16 M (Pfam)	114,45
EPY20843.1	insulysin			Metallopeptidase	Peptidase M16, M16 C e M16 M (Pfam)	118,71
EPY28550.1	insulysin			Metallopeptidase	Peptidase M16 M (Pfam)	94,82
EPY25077.1	insulysin			Metallopeptidase	Peptidase M16, M16 C e M16 M (Pfam)	92,74
EPY34371.1	mitochondrial processing peptide beta subunit			Metallopeptidase	Peptidase M16 e M16 C (Pfam)	46,8
EPY34191.1	mitochondrial processing peptide beta subunit			Metallopeptidase	Peptidase M16 e M16 C (Pfam)	55,9
EPY33142.1	cytosol alanyl aminopeptidase	MER0816483	M1	Metallopeptidase	Peptidase M1 e ERAP1 C (Pfam)	86,88
EPY23119.1	cytosol alanyl aminopeptidase	MER0813023	M1	Metallopeptidase	Peptidase M1 e ERAP1 C (Pfam)	95,86
EPY31979.1	amidohydrolase			Metallopeptidase	Peptidase M20 (Pfam)	31,99
EPY26040.1	amidohydrolase	MER0841009	M20D	Metallopeptidase	Peptidase M20 (Pfam)	31,99
EPY27037.1	amidohydrolase			Metallopeptidase	Peptidase M20 (Pfam)	44,31
EPY23618.1	amidohydrolase			Metallopeptidase	Peptidase M20 (Pfam)	44,31
EPY31250.1	STE24 endopeptidase	MER0883796	M48	Metallopeptidase	Peptidase M48 N e M48 (Pfam)	50,84
EPY30084.1	STE24 endopeptidase			Metallopeptidase	Peptidase M48 N e M48 (Pfam)	50,84
EPY28216.1	STE24 endopeptidase			Metallopeptidase	Peptidase M48 N e M48 (Pfam)	50,84
EPY23569.1	STE24 endopeptidase			Metallopeptidase	Peptidase M48 N e M48 (Pfam)	50,84
EPY19689.1	STE24 endopeptidase			Metallopeptidase	Peptidase M48 N e M48 (Pfam)	50,84
EPY36895.1	STE24 endopeptidase			Metallopeptidase	Peptidase M48 (Pfam)	25,82
EPY23619.1	metallo-peptidase			Metallopeptidase	Peptidase M1 (Pfam)	59,69
EPY19480.1	metallo-peptidase			Metallopeptidase	Peptidase M1 e ERAP1 C (Pfam)	83,97
EPY34173.1	metallo-peptidase			Metallopeptidase	zf-C6H2 e Peptidase M24 (Pfam)	44,57
EPY22108.1	metallo-peptidase	MER0814296	M1	Metallopeptidase	Peptidase M1 e ERAP1 C (Pfam)	96,47
EPY21346.1	metallo-peptidase	MER0820211	M3A	Metallopeptidase	Peptidase M3 (Pfam)	77,42

GenBank ID	Name	MEROPS ID	Family / Subfamily	Enzymatic class	Domains	Predict molecular mass (kDa)
EPY16032.1	metallo-peptidase			Metallopeptidase	Peptidase M14 (Pfam)	52,71
EPY30945.1	acetylornithine deacetylase	MER0729627	M20D	Metallopeptidase	Peptidase M20 (Pfam)	32,83
AGT02402.1	acetylornithine deacetylase	MER0842428	M20D	Metallopeptidase	Peptidase M20 Dimer (Pfam)	27,89
AGT02401.1	acetylornithine deacetylase			Metallopeptidase	Peptidase M20 Dimer (Pfam)	28,67
EPY19099.1	acetylornithine deacetylase	MER0840369	M20D	Metallopeptidase	Peptidase M20 (Pfam)	35,02
EPY33558.1	acetylornithine deacetylase			Metallopeptidase	Peptidase M20 Dimer (Pfam)	32,39
EPY33480.1	acetylornithine deacetylase			Metallopeptidase	Peptidase M20 Dimer (Pfam)	25,08
EPY36375.1	acetylornithine deacetylase	MER0850891	M20D	Metallopeptidase	Peptidase M20 (Pfam)	43,37
EPY32206.1	acetylornithine deacetylase			Metallopeptidase	Peptidase M20 Dimer (Pfam)	32,43
EPY20166.1	acetylornithine deacetylase	MER0854997	M20F	Metallopeptidase	Peptidase M20 Dimer (Pfam)	46,16
EPY19324.1	acetylornithine deacetylase	MER0933478	M20B	Metallopeptidase	Peptidase M20 Dimer (Pfam)	32,6
EPY37028.1	mitochondrial ATP-dependent zinc metallopeptidase	MER0876623	M41	Metallopeptidase	AAA e Peptidase M41 (Pfam)	54,49
EPY32991.1	ATP-dependent metalloprotease	MER0877945	M41	Metallopeptidase	AAA e Peptidase M41 (Pfam)	60,06
EPY34387.1	CGI-146 protein	MER0746913	C97	Cysteine peptidase	Peptidase C 97 (Pfam)	22,91
EPY31826.1	dipeptidyl-peptidase 9	MER0998766	S9B	Serine peptidase	DPPIV N e Peptidase S9 (Pfam)	100,56
EPY37245.1	ubiquitin carboxyl-terminal hydrolase			Cysteine peptidase	Peptidase C12 (Pfam)	36,09
EPY37047.1	ubiquitin carboxyl-terminal hydrolase L5			Cysteine peptidase	Peptidase C12 (Pfam)	27,99
EPY30748.1	ubiquitin carboxyl-terminal hydrolase L3	MER0708422	C12	Cysteine peptidase	Peptidase C12 (Pfam)	23,37
EPY29629.1	ubiquitin carboxyl-terminal hydrolase L3			Cysteine peptidase	Peptidase C12 (Pfam)	25,58
EPY29267.1	ubiquitin carboxyl-terminal hydrolase L3			Cysteine peptidase	Peptidase C12 (Pfam)	25,58
EPY25823.1	ubiquitin carboxyl-terminal hydrolase 14			Cysteine peptidase	UCH (Pfam)	38,68
EPY25161.1	ubiquitin carboxyl-terminal hydrolase L5	MER0708323	C12	Cysteine peptidase	Peptidase C12 (Pfam)	36,09
EPY24480.1	ubiquitin carboxyl-terminal hydrolase L3	MER0708395	C12	Cysteine peptidase	Peptidase C12 (Pfam)	14,34
EPY24373.1	ubiquitin carboxyl-terminal hydrolase 12/46	MER0712565	C19	Cysteine peptidase	UCH (Pfam)	54,57
EPY24079.1	ubiquitin carboxyl-terminal hydrolase 7			Cysteine peptidase	UCH (Pfam)	102,6
EPY20791.1	ubiquitin carboxyl-terminal hydrolase 14	MER0710863	C19	Cysteine peptidase	UBQ (SMART - Interpro) e UCH (Pfam)	49,97
EPY20643.1	ubiquitin carboxyl-terminal hydrolase 7	MER0711550	C19	Cysteine peptidase	UCH (Pfam)	102,6
EPY19892.1	ubiquitin carboxyl-terminal hydrolase 5/13	MER0708668	C19	Cysteine peptidase	2xZnF_UBP (SMART) e UCH (Pfam)	79,42
EPY19597.1	ubiquitin carboxyl-terminal hydrolase L3	MER0707603	C12	Cysteine peptidase	Peptidase C12 (Pfam)	25,58
EPY19321.1	ubiquitin carboxyl-terminal hydrolase 5/13	MER0710980	C19	Cysteine peptidase	2xZnF_UBP (SMART) e UCH (Pfam)	68,96
EPY18793.1	ubiquitin carboxyl-terminal hydrolase 14			Cysteine peptidase	UCH (Pfam)	38,73
EPY18567.1	ubiquitin carboxyl-terminal hydrolase 7	MER0714279	C19	Cysteine peptidase	UCH e USP7_ICP0 bdg (Pfam)	112,13
EPY34216.1	presenilin 1			Aspartic peptidase	PSN (Smart)	38,07
EPY31463.1	presenilin 1	MER0694331	A22A	Aspartic peptidase	PSN (Smart)	38,07
EPY24997.1	presenilin 1			Aspartic peptidase	PSN (Smart)	23,4
EPY21110.1	presenilin 1			Aspartic peptidase	PSN (Smart)	38,07
EPY29661.1	minor histocompatibility antigen H13			Aspartic peptidase	PSN (Smart)	38,26
EPY19633.1	minor histocompatibility antigen H13	MER0481363	A22B	Aspartic peptidase	PSN (Smart)	42,26
EPY28608.1	Xaa-Pro dipeptidase			Metallopeptidase	AMP_N (SMART) e Peptidase M24	54,67
EPY22915.1	Xaa-Pro dipeptidase	MER1245372	M24B	Metallopeptidase	AMP_N (SMART) e Peptidase M24	54,31
EPY20296.1	Xaa-Pro dipeptidase			Metallopeptidase	AMP_N (SMART) e Peptidase M24	53,54
EPY27848.1	ATP-dependent zinc metallopeptidase	MER0876087	M41	Metallopeptidase	AAA e Peptidase M41 (Pfam)	43,04
EPY26176.1	ATP-dependent zinc metallopeptidase	MER0877647	M41	Metallopeptidase	AAA e Peptidase M41 (Pfam)	52,53
EPY20591.1	ATP-dependent zinc metallopeptidase	MER0877586	M41	Metallopeptidase	AAA e Peptidase M41 (Pfam)	77,33
EPY18894.1	ATP-dependent zinc metallopeptidase	MER0875473	M41	Metallopeptidase	AAA e Peptidase M41 (Pfam)	61,73
EPY25542.1	metallopeptidase	MER0870540	M41	Metallopeptidase	AAA e Peptidase M41 (Pfam)	64,13
EPY24568.1	cell division protease FtsH			Metallopeptidase	AAA e Peptidase M41 (Pfam)	68,76
EPY23082.1	PAB-dependent poly(A)-specific ribonuclease subunit 2			Cysteine peptidase	UCH_1 (Pfam) e EXOIII (Smart)	121,1
EPY20833.1	PAB-dependent poly(A)-specific ribonuclease subunit 2			Cysteine peptidase	UCH_1 (Pfam) e EXOIII (Smart)	124,1
EPY19197.1	PAB-dependent poly(A)-specific ribonuclease subunit 2			Cysteine peptidase	UCH_1 (Pfam) e EXOIII (Smart)	110,2
EPY18858.1	prolyl oligopeptidase	MER0993707	S9A	Serine peptidase	Peptidase S9_N e Peptidase S9 (Pfam)	78,19
EPY17974.1	oligopeptidase B	MER0991251	S9A	Serine peptidase	Peptidase S9_N e Peptidase S9 (Pfam)	69,55
EPY37176.1	thimet oligopeptidase			Metallopeptidase	Peptidase M3 (Pfam)	70,85
EPY21849.1	thimet oligopeptidase	MER0817250	M3A	Metallopeptidase	Peptidase M3 (Pfam)	76,96
EPY19515.1	thimet oligopeptidase			Metallopeptidase	Peptidase M3 (Pfam)	54,51
EPY22043.1	Xaa-Pro aminopeptidase			Metallopeptidase	Creatinase N_2, Peptidase M24 e Peptidase M24_C (Pfam)	53,53
EPY21873.1	AFG3 family protein	MER0867373	M41	Metallopeptidase	AAA e Peptidase M41 (Pfam)	82,24
AGT02400.1	aminoacylase	MER0847027	M20D	Metallopeptidase	Peptidase M20 (Pfam)	44,31
EPY37270.1	leishmanolysin	MER0824176	M8	Metallopeptidase	Peptidase M8 (Pfam)	58,34
EPY36381.1	leishmanolysin	MER0824173	M8	Metallopeptidase	Peptidase M8 (Pfam)	47,93
EPY23648.1	leishmanolysin	MER0824173	M8	Metallopeptidase	Peptidase M8 (Pfam)	66,31

GenBank ID	Name	MEROPS ID	Family / Subfamily	Enzymatic class	Domains	Predict molecular mass (kDa)
EPY23056.1	leishmanolysin			Metallopeptidase	Peptidase M8 (Pfam)	48,09
EPY20885.1	leishmanolysin	MER0824394	M8	Metallopeptidase	Peptidase M8 (Pfam)	58,66
EPY19626.1	leishmanolysin			Metallopeptidase	Peptidase M8 (Pfam)	33,01
EPY19508.1	leishmanolysin			Metallopeptidase	Peptidase M8 (Pfam)	36,59
EPY32300.1	leishmanolysin-like	MER0824163	M8	Metallopeptidase	Peptidase M8 (Pfam)	27,56
EPY36134.1	serine carboxypeptidase III precursor	MER0943872	S10	Serine peptidase	Peptidase S10 (Pfam)	56,57
EPY35944.1	methionyl aminopeptidase			Metallopeptidase	Peptidase M24 (Pfam)	24,95
EPY32683.1	methionyl aminopeptidase			Metallopeptidase	Peptidase M24 (Pfam)	47,73
EPY21878.1	methionyl aminopeptidase			Metallopeptidase	Peptidase M24 (Pfam)	51,8
EPY34685.1	cathepsin L			Cysteine peptidase	Inhibithor I29 e Pept C1 (Smart)	42,86
EPY25881.1	cathepsin L	MER0704102	C1A	Cysteine peptidase	Inhibithor I29 e Pept C1 (Smart)	42,86
EPY33340.1	carboxypeptidase Taq			Metallopeptidase	2xPeptidase M32 (Pfam)	35,38
EPY25743.1	carboxypeptidase Taq			Metallopeptidase	Peptidase M32 (Pfam)	38,95
EPY23120.1	carboxypeptidase Taq	MER0858053	M32.005	Metallopeptidase	2xPeptidase M32 (Pfam)	52,98
EPY22775.1	carboxypeptidase Taq	MER0857611	M32.005	Metallopeptidase	Peptidase M32 (Pfam)	48,01
EPY30718.1	aspartyl aminopeptidase			Metallopeptidase	Peptidase M18 (Pfam)	26,34
EPY30100.1	metacaspase			Cysteine peptidase	Peptidase C14 (Pfam)	36,78
EPY27857.1	metacaspase			Cysteine peptidase	Peptidase C14 (Pfam)	46,77
EPY23392.1	metacaspase			Cysteine peptidase	Peptidase C14 (Pfam)	24,11
EPY21874.1	metacaspase 5			Cysteine peptidase	Peptidase C14 e Atg8 (Pfam)	67,77
EPY28611.1	cathepsin A	MER0946251	S10	Serine peptidase	Peptidase S10 (Pfam)	25,79
EPY28297.1	cathepsin A			Serine peptidase	Peptidase S10 (Pfam)	37,23
EPY24936.1	peptidyl-dipeptidase Dcp			Metallopeptidase	Peptidase M3 (Pfam)	77,92
EPY23466.1	peptidyl-dipeptidase Dcp	MER0819681	M3A	Metallopeptidase	Peptidase M3 (Pfam)	77,92
EPY24291.1	aminopeptidase	MER0813353	M1	Metallopeptidase	Peptidase M1 (Pfam)	59,47
EPY23974.1	aminopeptidase			Metallopeptidase	Peptidase M17 (Pfam)	60,54
EPY22227.1	aminopeptidase			Metallopeptidase	Peptidase M17 (Pfam)	22,97
EPY21441.1	leucyl aminopeptidase	MER0929830	M17	Metallopeptidase	Peptidase M17 (Pfam)	37,75
EPY24088.1	leucyl aminopeptidase			Metallopeptidase	Peptidase M17 (Pfam)	37,75
EPY17122.1	hypothetical protein STCU_10802			Cysteine peptidase	UCH (Pfam)	111,98
EPY37240.1	hypothetical protein STCU_00057			Metallopeptidase	Peptidase M76 (Pfam)	34,24
EPY37213.1	hypothetical protein STCU_00084			Cysteine peptidase	Peptidase C54 (Pfam)	39,99
EPY36600.1	hypothetical protein STCU_00500				Peptidase M48 (Pfam)	10,48
EPY35858.1	hypothetical protein STCU_00876			Metallopeptidase	Peptidase M76 (Pfam)	34,24
EPY32838.1	hypothetical protein STCU_02617				DUF2012 (Pfam)	11,02
EPY29522.1	hypothetical protein STCU_04499					49,01
EPY29154.1	hypothetical protein STCU_04698			Metallopeptidase	Peptidase M76 (Pfam)	34,24
EPY28259.1	hypothetical protein STCU_05221				DUF2012 (Pfam)	25,39
EPY28034.1	hypothetical protein STCU_05334					14.17
EPY27250.1	hypothetical protein STCU_05845			Cysteine peptidase	Peptidase C97 (Pfam)	68,9
EPY26994.1	hypothetical protein STCU_05975	MER0709054	C19	Cysteine peptidase	UCH (Pfam)	33,21
EPY26293.1	hypothetical protein STCU_06224			Cysteine peptidase	Peptidase C54 (Pfam)	42,01
EPY24462.1	hypothetical protein STCU_07168			Cysteine peptidase	Peptidase C97 (Pfam)	43,67
EPY22827.1	hypothetical protein STCU_08108	MER0908088	M76	Metallopeptidase	Peptidase M76 (Pfam)	34,24
EPY20856.1	hypothetical protein STCU_08801	MER0729507	C54	Cysteine peptidase	Peptidase C54 (Pfam)	42,01
EPY19991.1	hypothetical protein STCU_09204	MER0746994	C97	Cysteine peptidase	Peptidase C97 (Pfam)	68,9
EPY18820.1	hypothetical protein STCU_09758			Metallopeptidase	Peptidase M64 (Pfam)	105,56
EPY15214.1	hypothetical protein STCU_12241	MER0709240	C19	Cysteine peptidase	UCH (Pfam)	31,91
EPY36438.1	hypothetical protein STCU_00582				DUF2817 (Pfam)	43,02
EPY34325.1	hypothetical protein STCU_01645				DUF2817 (Pfam)	45
EPY32769.1	hypothetical protein STCU_02668				DUF2817 (Pfam)	45
EPY31789.1	hypothetical protein STCU_03236			Metallopeptidase	Peptidase M18 (Pfam)	36,4
EPY31032.1	hypothetical protein STCU_03671				zf-MIZ (Pfam)	25,51
EPY27491.1	hypothetical protein STCU_05724				DUF1796 (Pfam)	40,32
EPY25215.1	hypothetical protein STCU_06785				DUF2817 (Pfam)	45
EPY25048.1	hypothetical protein STCU_06869				DUF1796 (Pfam)	43,74
EPY23065.1	hypothetical protein STCU_07893			Metallopeptidase	Peptidase M18 (Pfam)	36,4
EPY23002.1	hypothetical protein STCU_07956				DUF1796 (Pfam)	51,25
EPY21158.1	hypothetical protein STCU_08671				DUF1796 (Pfam)	43,74
EPY19275.1	hypothetical protein STCU_09545				CRAL_TRIO (Pfam)	91,45
EPY19118.1	hypothetical protein STCU_09608			Serine peptidase	Peptidase S9 (Pfam)	31,14
EPY18797.1	hypothetical protein STCU_09769				CRAL_TRIO (Pfam)	121,69
EPY17954.1	hypothetical protein STCU_10292			Metallopeptidase	Peptidase M16 (Pfam)	12,74
EPY17737.1	hypothetical protein STCU_10437	MER0824326	M8	Metallopeptidase	2xPeptidase M8 (Pfam)	131,64

GenBank ID	Name	MEROPS ID	Family / Subfamily	Enzymatic class	Domains	Predict molecular mass (kDa)
EPY17617.1	hypothetical protein STCU_10514	MER0978508	S8A	Serine peptidase	Peptidase S8 (Pfam)	70,91
EPY16968.1	hypothetical protein STCU_10885			Metallopeptidase	Peptidase M14 (Pfam)	69,14
EPY16921.1	hypothetical protein STCU_10910			Metallopeptidase	Peptidase M14 (Pfam)	26,21
EPY15090.1	hypothetical protein STCU_12352	MER0844014	M20D	Metallopeptidase	Peptidase M20 (Pfam)	15,96
EPY27912.1	ubiquitin hydrolase	MER0714357	C19	Cysteine peptidase	UCH (Pfam)	51,33
EPY18313.1	ubiquitin thiolesterase	MER0709920	C19	Cysteine peptidase	UCH (Pfam)	41,73
EPY20701.1	insulysin			Metallopeptidase	Peptidase M16 M (Pfam)	94,72
EPY26850.1	hypothetical protein STCU_06047	MER0962738	C115	Cysteine peptidase	MINDY_DUB (Pfam)	55,68
EPY30738.1	hypothetical protein STCU_06047			Cysteine peptidase	MINDY_DUB (Pfam)	55,68
EPY37265.1	hypothetical protein STCU_06047			Cysteine peptidase	MINDY_DUB (Pfam)	55,68
EPY15728.1	COP9 signalosome complex subunit 5	MER0907441	M67A	Metallopeptidase	JAB_MPN (Smart - Interpro)	34,71
EPY19398.1	oligopeptidase B protein	MER0993440	S9A	Serine peptidase	Peptidase S9 (Pfam)	65,21
EPY19493.1	hypothetical protein STCU_09427	MER0707286	C2A	Cysteine peptidase	CysPc (SMART - Interpro)	37,84
EPY22192.1	calpain-like protein	MER0707092	C2A	Cysteine peptidase	DUF1935 (Pfam) e CysPc (SMART - Interpro)	94,15
EPY20729.1	phosphatidylinositol glycan, class K	MER0685858	C13	Cysteine peptidase	Peptidase C13 (Pfam)	38,49
EPY21463.1	ubiquitin thioesterase protein OTUB1	MER0735108	C65	Cysteine peptidase	Peptidase C65 (Pfam)	34,27
EPY25227.1	lysosomal/endosomal membrane protein p67	MER0745547	C95	Cysteine peptidase	Phospholip_B (Pfam)	85,01
EPY20654.1	20S proteasome subunit beta 5	MER1094004	T1A	Threonine peptidase	Proteasome (Pfam)	34,22
EPY20907.1	20S proteasome subunit beta 5			Threonine peptidase	Proteasome (Pfam)	34,22
EPY25672.1	20S proteasome subunit beta 5			Threonine peptidase	Proteasome (Pfam)	34,22
WP_015237866.1	ATP-dependent metallopeptidase FtsH/Yme1/Tma	MER0876136	M41	Metallopeptidase	AAA e Peptidase M41 (Pfam)	68,76
EPY25738.1	20S proteasome subunit alpha 4	MER1090173	T1A	Threonine peptidase	Proteasome_A_N (Smart) e Proteasome (Pfam)	27,63
EPY26713.1	20S proteasome subunit alpha 4			Threonine peptidase	Proteasome_A_N (Smart) e Proteasome (Pfam)	27,63
EPY37138.1	20S proteasome subunit alpha 4			Threonine peptidase	Proteasome_A_N (Smart) e Proteasome (Pfam)	27,63
EPY26964.1	20S proteasome subunit beta 3	MER1091162	T1A	Threonine peptidase	Proteasome (Pfam)	22,58
EPY35678.1	proteasome beta 3 subunit			Threonine peptidase	Proteasome (Pfam)	22,58
EPY37293.1	proteasome beta 3 subunit			Threonine peptidase	Proteasome (Pfam)	22,58
EPY28527.1	20S proteasome subunit beta 4	MER1088090	T1A	Threonine peptidase	Proteasome (Pfam)	22,96
EPY30424.1	DNA damage-inducible protein 1	MER0699875	A28A	Aspartic peptidase	Asp_protease e UBA_4 (Pfam)	32,35
EPY33263.1	aminopeptidase	MER0931177	M18	Metallopeptidase	Peptidase M18 (Pfam)	46,75
EPY33037.1	20S proteasome subunit beta 3	MER1093061	T1A	Threonine peptidase	Proteasome (Pfam)	12,21
EPY33242.1	proteasome alpha 3 subunit	MER1093513	T1A	Threonine peptidase	Proteasome (Pfam)	37,76
EPY33812.1	proteasome alpha 1 subunit	MER1092492	T1A	Threonine peptidase	Proteasome_A_N (Smart) e Proteasome (Pfam)	29,85
EPY36996.1	20S proteasome subunit alpha 6			Threonine peptidase	Proteasome_A_N (Smart) e Proteasome (Pfam)	29,85
EPY31808.1	calpain family cysteine protease-like protein	MER0705988	C2A	Cysteine peptidase	DUF1935 (Pfam) e CysPc (SMART - Interpro)	80,9
EPY34767.1	20S proteasome subunit alpha 5	MER1088920	T1A	Threonine peptidase	Proteasome (Pfam)	15,28
EPY33029.1	BRCA1/BRCA2-containing complex subunit 3	MER0906657	M67A	Metallopeptidase	JAB (Pfam)	23,24
EPY33309.1	26S proteasome regulatory subunit N11	MER0907700	M67A	Metallopeptidase	JAB_MPN (SMART - Interpro)	29
EPY37252.1	calpain family cysteine protease-like protein	MER0706801	C2A	Cysteine peptidase	DUF1935 (Pfam) e CysPc (SMART - Interpro)	79,93
EPY35928.1	20S proteasome subunit beta 6	MER1091416	T1A	Threonine peptidase	Proteasome (Pfam)	13,67
EPY22196.1	calpain-like protein	MER0706481	C2A	Cysteine peptidase	CysPc (SMART - Interpro)	95,16
EPY37099.1	proteasome beta-1 subunit	MER1089825	T1A	Threonine peptidase	Proteasome (Pfam)	31,05
EPY24506.1	calpain-7	MER0706044	C2A	Cysteine peptidase	CysPc (SMART - Interpro)	84,89
EPY21015.1	calpain-like protein	MER0706249	C2A	Cysteine peptidase	CysPc e Calpain_III (SMART - Interpro)	80,55
EPY21075.1	hypothetical protein STCU_08709	MER0705984	C2A	Cysteine peptidase	Peptidase_C2 (Pfam)	44,89
EPY23620.1	hypothetical protein STCU_07620	MER0706600	C2A	Cysteine peptidase	CysPc (SMART - Interpro) e Calpain_III (Pfam)	35,1
EPY28641.1	BRCA1/BRCA2-containing complex subunit 3	MER0905902	M67A	Metallopeptidase	JAB (SMART - Interpro)	32,35
EPY29006.1	hypothetical protein STCU_04772	MER0706873	C2A	Cysteine peptidase	CysPc (SMART - Interpro)	28,42
EPY30456.1	26S proteasome regulatory subunit N11	MER0905266	M67A	Metallopeptidase	JAB (SMART - Interpro)	39,37
EPY23074.1	aminopeptidase	MER0930945	M18	Metallopeptidase	Peptidase M18 (Pfam)	49,11
EPY23710.1	metallo-peptidase, Clan MH, Family M20			Metallopeptidase	Peptidase M18 (Pfam)	49,11

GenBank ID	Name	MEROPS ID	Family / Subfamily	Enzymatic class	Domains	Predict molecular mass (kDa)
EPY26323.1	aminopeptidase			Metallopeptidase	Peptidase M18 (Pfam)	49,11
EPY28575.1	metallo-peptidase, Clan MH, Family M20			Metallopeptidase	Peptidase M18 (Pfam)	49,11
EPY34301.1	20S proteasome subunit alpha 6	MER1093813	T1A	Threonine peptidase	Proteasome (Pfam)	21,67
EPY34446.1	dipeptidase E	MER1055581	S51	Serine peptidase	Peptidase_S51 (Pfam)	37,39
EPY35086.1	20S proteasome subunit beta 2	MER1089573	T1A	Threonine peptidase	Proteasome (Pfam)	27,79
EPY36921.1	20S proteasome subunit beta 2	MER1091426	T1A	Threonine peptidase	Proteasome (Pfam)	27,77
EPY37086.1	proteasome alpha 3 subunit	MER1090752	T1A	Threonine peptidase	Proteasome (Pfam)	37,77
EPY37236.1	serine carboxypeptidase S28	MER1054906	S28	Serine peptidase	Peptidase_S28 (Pfam)	55,48
EPY34270.1	20S proteasome subunit beta 3	MER1088955	T1A	Threonine peptidase	Proteasome (Pfam)	17,91
EPY35012.1	20S proteasome subunit beta 3			Metallopeptidase	JAB_MPN (Smart - Interpro) e MitMem_reg (Pfam)	40,93
EPY35643.1	20S proteasome subunit alpha 7			Threonine peptidase	Proteasome (Pfam)	17,46
EPY31083.1	20S proteasome subunit alpha 3			Threonine peptidase	Proteasome_A_N (Smart) e Proteasome (Pfam)	32,06
EPY30848.1	20S proteasome subunit alpha 1			Threonine peptidase	Proteasome (Pfam)	14,76
EPY31564.1	20S proteasome subunit beta 1			Threonine peptidase	Proteasome (Pfam)	26,45
EPY27906.1	20S proteasome subunit alpha 5			Threonine peptidase	Proteasome_A_N (Smart) e Proteasome (Pfam)	33,25
EPY27567.1	20S proteasome subunit beta 7			Threonine peptidase	Proteasome (Pfam)	24,86
EPY27296.1	20S proteasome subunit alpha 7			Threonine peptidase	Proteasome_A_N (Smart) e Proteasome (Pfam)	25,63
EPY26142.1	20S proteasome subunit beta 6			Threonine peptidase	Proteasome (Pfam)	27,79
EPY25594.1	20S proteasome subunit beta 1			Threonine peptidase	Proteasome (Pfam)	16,05
EPY20661.1	20S proteasome subunit alpha 2			Threonine peptidase	Proteasome_A_N (Smart) e Proteasome (Pfam)	25,21
EPY37253.1	hypothetical protein STCU_00044			Serine peptidase	Trypsin_2 (Pfam)	21,41
EPY28926.1	hypothetical protein STCU_04812			Cysteine peptidase	2xZnF_RBZ (Smart - Interpro) e OTU (Pfam)	63,46
EPY26731.1	ubiquitin thioesterase protein OTUB1			Cysteine peptidase	Peptidase_C65 (Pfam)	29,77
EPY26615.1	hypothetical protein STCU_06166			Cysteine peptidase	2xZnF_RBZ (Smart - Interpro) e OTU (Pfam)	63,46
EPY25099.1	ubiquitin thioesterase protein OTUB1			Cysteine peptidase	Peptidase_C65 (Pfam)	23,54
EPY20222.1	hypothetical protein STCU_09103			Cysteine peptidase	Peptidase_C65 (Pfam)	61,66
EPY27146.1	rhomboid-like protein			Serine peptidase	Rhomboid (Pfam)	38,04
EPY31842.1	20S proteasome subunit beta 4			Threonine peptidase	Proteasome (Pfam)	22,96
EPY37280.1	proteasome regulatory subunit N8			Metallopeptidase	JAB_MPN (Smart - Interpro) e MitMem_reg (Pfam)	40,93
EPY30482.1	20S proteasome subunit beta 7			Threonine peptidase	Proteasome (Pfam)	24,86
EPY36015.1	20S proteasome subunit beta 7			Threonine peptidase	Proteasome (Pfam)	24,86
EPY33732.1	20S proteasome subunit alpha 7			Threonine peptidase	Proteasome_A_N (Smart) e Proteasome (Pfam)	25,63
EPY29669.1	20S proteasome subunit beta 6			Threonine peptidase	Proteasome (Pfam)	27,79
EPY32649.1	20S proteasome subunit beta 1			Threonine peptidase	Proteasome (Pfam)	16,05
EPY22189.1	proteasome alpha 2 subunit			Threonine peptidase	Proteasome_A_N (Smart) e Proteasome (Pfam)	25,21
EPY25698.1	20S proteasome subunit alpha 2			Threonine peptidase	Proteasome_A_N (Smart) e Proteasome (Pfam)	25,21
EPY27541.1	proteasome alpha 2 subunit			Threonine peptidase	Proteasome_A_N (Smart) e Proteasome (Pfam)	25,21
EPY33174.1	20S proteasome subunit alpha 2			Threonine peptidase	Proteasome_A_N (Smart) e Proteasome (Pfam)	25,21
EPY27615.1	aminopeptidase			Metallopeptidase	Peptidase_M24 (Pfam)	49,3

TABLE III

Differential expressed peptidases from wild-type (WT) and aposymbiotic (APO) *Strigomonas culicis* detected in Brunoro's proteome[10] and Bombaça's proteome[11]

					Brunoro's proteome[10]		Bombaça's proteome[11]		
GenBank ID	MEROPS	Name	Enzymatic class	Domains	WT	APO	WT	APO	Note
EPY28464.1		serine peptidase	Serine peptidase	Rhomboid (Pfam)	(=)	(=)			
EPY31826.1	MER0998766	dipeptidyl-peptidase 9	Serine peptidase	DPPIV N e Peptidase S9 (Pfam)	(=)	(=)			
EPY18858.1	MER0993707	prolyl oligopeptidase	Serine peptidase	Peptidase S9_N e Peptidase S9 (Pfam)	(=)	(=)			
EPY17974.1	MER0991251	oligopeptidase B	Serine peptidase	Peptidase S9_N e Peptidase S9 (Pfam)			(=)	(=)	
EPY34446.1	MER1055581	dipeptidase E	Serine peptidase	Peptidase_S51 (Pfam)	(-)	(+)			
EPY33248.1	MER1089424	ATP-dependent HslUV protease	Threonine peptidase	Proteasome (Pfam)	(=)	(=)			
EPY24218.1	MER1091871	ATP-dependent HslUV protease	Threonine peptidase	Proteasome (Pfam)	(+)	(-)			
EPY20654.1	MER1094004	20S proteasome subunit beta 5	Threonine peptidase	Proteasome (Pfam)	(=)	(=)	(=)	(=)	
EPY25738.1	MER1090173	20S proteasome subunit alpha 4	Threonine peptidase	Proteasome_A_N (Smart) e Proteasome (Pfam)	(=)	(=)	(-)	(+)	
EPY26964.1	MER1091162	20S proteasome subunit beta 3	Threonine peptidase	Proteasome (Pfam)	(=)	(=)	(i)	(+++)	
EPY33242.1	MER1093513	proteasome alpha 3 subunit	Threonine peptidase	Proteasome (Pfam)	(=)	(=)			
EPY33812.1	MER1092492	proteasome alpha 1 subunit	Threonine peptidase	Proteasome_A_N (Smart) e Proteasome (Pfam)	(=)	(=)	(=)	(=)	
EPY37099.1	MER1089825	proteasome beta-1 subunit	Threonine peptidase	Proteasome (Pfam)	(=)	(=)			
EPY34301.1	MER1093813	20S proteasome subunit alpha 6	Threonine peptidase	Proteasome (Pfam)	(=)	(=)			
EPY35086.1	MER1089573	20S proteasome subunit beta 2	Threonine peptidase	Proteasome (Pfam)	(=)	(=)			
EPY36921.1	MER1091426	20S proteasome subunit beta 2	Threonine peptidase	Proteasome (Pfam)	(=)	(=)			
EPY37086.1	MER1090752	proteasome alpha 3 subunit	Threonine peptidase	Proteasome (Pfam)	(=)	(=)			
EPY31083.1		20S proteasome subunit alpha 3	Threonine peptidase	Proteasome_A_N (Smart) e Proteasome (Pfam)	(=)	(=)			
EPY30848.1		20S proteasome subunit alpha 1	Threonine peptidase	Proteasome (Pfam)			(=)	(=)	
EPY27906.1		20S proteasome subunit alpha 5	Threonine peptidase	Proteasome_A_N (Smart) e Proteasome (Pfam)	(=)	(=)	(=)	(=)	
EPY27567.1		20S proteasome subunit beta 7	Threonine peptidase	Proteasome (Pfam)			(=)	(=)	
EPY27296.1		20S proteasome subunit alpha 7	Threonine peptidase	Proteasome_A_N (Smart) e Proteasome (Pfam)	(=)	(=)	(=)	(=)	
EPY26142.1		20S proteasome subunit beta 6	Threonine peptidase	Proteasome (Pfam)	(=)	(=)	(=)	(=)	
EPY20661.1		20S proteasome subunit alpha 2	Threonine peptidase	Proteasome_A_N (Smart) e Proteasome (Pfam)	(=)	(=)	(=)	(=)	
EPY23907.1	MER0704373	cysteine peptidase C	Cysteine peptidase	Pept_C1 (SMART - Interpro)	(+)	(-)	(=)	(=)	
EPY26229.1		cysteine peptidase	Cysteine peptidase	ZnF_UBP e UBA (SMART - Interpro)	(+)	(-)			
EPY34688.1	MER0707153	cysteine peptidase	Cysteine peptidase	DUF1935 (Pfam) e CysPc (SMART - Interpro)	(=)	(=)			
EPY31483.1	MER0706650	cysteine peptidase	Cysteine peptidase	DUF1935 (Pfam) e CysPc (SMART - Interpro)	(=)	(=)	(i)	(+++)	
EPY18663.1		cysteine peptidase	Cysteine peptidase	2xRPT1 (Prospero)	(=)	(=)	(-)	(+)	Non-proteolytic active domain
EPY30438.1		inhibitor of cysteine peptidase	Cysteine peptidase	Inhibitor I42 Domain	(+)	(-)			Non-proteolytic active domain
EPY22055.1		cysteine peptidase A	Cysteine peptidase	Inhibitor I29 e Pept_C1 (SMART - Interpro), e DUFF 3586 (Pfam)	(=)	(=)			
EPY21129.1	MER0702575	cysteine peptidase A	Cysteine peptidase	Inhibitor I29 e Pept_C1 (SMART - Interpro), e DUFF 3586 (Pfam)	(=)	(=)			
EPY36883.1		calpain-like cysteine peptidase	Cysteine peptidase	DUF1935 (Pfam)	(=)	(=)			Non-proteolytic active domain
EPY34294.1		calpain-like cysteine peptidase	Cysteine peptidase	DUF1935 (Pfam)	(=)	(=)			Non-proteolytic active domain
EPY33460.1		calpain-like cysteine peptidase	Cysteine peptidase	DUF1935 (Pfam)	(+)	(-)			Non-proteolytic active domain
EPY31224.1		calpain-like cysteine peptidase	Cysteine peptidase	DUF1935 (Pfam)	(+++)	(i)	(i)	(+++)	Non-proteolytic active domain
EPY19819.1	MER0706686	calpain-like cysteine peptidase	Cysteine peptidase	DUF1935 (Pfam) e CysPc (SMART - Interpro)	(-)	(+)			
EPY30436.1	MER0706700	calpain-like cysteine peptidase	Cysteine peptidase	DUF1935 (Pfam) e CysPc (SMART - Interpro)	(=)	(=)			
EPY29273.1	MER0706231	calpain-like cysteine peptidase	Cysteine peptidase	DUF1935 (Pfam) e CysPc (SMART - Interpro)	(=)	(=)	(+)	(-)	
EPY29106.1		calpain-like cysteine peptidase	Cysteine peptidase	---	(=)	(=)			No domain identified
EPY28743.1		calpain-like cysteine peptidase	Cysteine peptidase	DUF1935 (Pfam) e CysPc (SMART - Interpro)	(-)	(+)			
EPY28675.1	MER0706847	calpain-like cysteine peptidase	Cysteine peptidase	DUF1935 (Pfam) e CysPc (SMART - Interpro)	(=)	(=)	(=)	(=)	
EPY22712.1	MER0706095	calpain-like cysteine peptidase	Cysteine peptidase	DUF1935 (Pfam) e CysPc (SMART - Interpro)	(=)	(=)			

					Brunoro's proteome[10]		Bombaça's proteome[11]		
GenBank ID	MEROPS	Name	Enzymatic class	Domains	WT	APO	WT	APO	Note
EPY20021.1	MER0707061	calpain-like cysteine peptidase	Cysteine peptidase	DUF1935 (Pfam) e CysPc (SMART - Interpro)	(+)	(-)			
EPY19437.1	MER0707143	calpain-like cysteine peptidase	Cysteine peptidase	DUF1935 (Pfam) e CysPc (SMART - Interpro)	(-)	(+)			
EPY19437.1		calpain-like cysteine peptidase	Cysteine peptidase	RPT1 (Propero) e CysPc (SMART - Interpro)	(+)	(-)	(-)	(+)	
EPY19174.1		calpain-like cysteine peptidase	Cysteine peptidase	2xRPT2 e RPT3 (Prospero)	(+)	(-)			Non-proteolytic active domain
EPY18674.1		calpain-like cysteine peptidase	Cysteine peptidase	2xRPT1 (Prospero)	(-)	(+)			Non-proteolytic active domain
EPY18665.1		calpain-like cysteine peptidase	Cysteine peptidase	RPT4 e RPT3 (Prospero)	(=)	(=)	(=)	(=)	Non-proteolytic active domain
EPY18645.1	MER0706922	calpain-like cysteine peptidase	Cysteine peptidase	RPT1 (Propero) e CysPc (SMART - Interpro)	(=)	(=)	(i)	(+++)	
EPY25161.1	MER0708323	ubiquitin carboxyl-terminal hydrolase L5	Cysteine peptidase	Peptidase C12 (Pfam)	(=)	(=)			
EPY20791.1	MER0710863	ubiquitin carboxyl-terminal hydrolase 14	Cysteine peptidase	UBQ (SMART - Interpro) e UCH (Pfam)	(=)	(=)			
EPY19892.1	MER0708668	ubiquitin carboxyl-terminal hydrolase 5/13	Cysteine peptidase	2xZnF_UBP (SMART) e UCH (Pfam)	(+)	(-)			
EPY19321.1	MER0710980	ubiquitin carboxyl-terminal hydrolase 5/13	Cysteine peptidase	2xZnF_UBP (SMART) e UCH (Pfam)	(+)	(-)			
EPY18793.1		ubiquitin carboxyl-terminal hydrolase 14	Cysteine peptidase	UCH (Pfam)	(=)	(=)			
EPY19493.1	MER0707286	hypothetical protein STCU_09427	Cysteine peptidase	CysPc (SMART - Interpro)	(=)	(=)			
EPY25227.1	MER0745547	lysosomal/endosomal membrane protein p67	Cysteine peptidase	Phospholip_B (Pfam)	(+)	(-)			
EPY24850.1	MER0922125	mitochondrial processing peptidase	Metallopeptidase	Peptidase M16 e Peptidase M16 C (Pfam)	(=)	(=)	(+++)	(i)	
EPY27707.1		mitochondrial processing peptidase	Metallopeptidase	Peptidase M16 (Pfam)	(=)	(=)	(+++)	(i)	
EPY35284.1		mitochondrial processing peptidase	Metallopeptidase	Peptidase M16 (Pfam)	(=)	(=)	(=)	(=)	
EPY34199.1		mitochondrial processing peptidase	Metallopeptidase	Peptidase M16 (Pfam)	(=)	(=)	(=)	(=)	
EPY27820.1		mitochondrial processing peptidase	Metallopeptidase	Peptidase M16 (Pfam)	(=)	(=)			
EPY23888.1	MER0932050	peptidase T	Metallopeptidase	Peptidase M28 (Pfam)			(=)	(=)	
EPY22990.1	MER0818143	mitochondrial intermediate peptidase	Metallopeptidase	Peptidase M3 (Pfam)	(-)	(+)			
EPY23119.1	MER0813023	cytosol alanyl aminopeptidase	Metallopeptidase	Peptidase M1 e ERAP1 C (Pfam)	(=)	(=)	(i)	(+++)	
EPY23618.1		amidohydrolase	Metallopeptidase	Peptidase M20 (Pfam)	(=)	(=)	(=)	(=)	
EPY34173.1		metallo-peptidase	Metallopeptidase	zf-C6H2 e Peptidase M24 (Pfam)	(i)	(+++)			
EPY21346.1	MER0820211	metallo-peptidase	Metallopeptidase	Peptidase M3 (Pfam)	(-)	(+)			
EPY20166.1	MER0854997	acetylornithine deacetylase	Metallopeptidase	Peptidase M20 Dimer (Pfam)	(-)	(+)			
EPY19324.1	MER0933478	acetylornithine deacetylase	Metallopeptidase	Peptidase M20 Dimer (Pfam)	(-)	(+)			
EPY26176.1	MER0877647	ATP-dependent zinc metallopeptidase	Metallopeptidase	AAA e Peptidase M41 (Pfam)	(-)	(+)	(=)	(=)	
EPY20591.1	MER0877586	ATP-dependent zinc metallopeptidase	Metallopeptidase	AAA e Peptidase M41 (Pfam)	(=)	(=)	(=)	(=)	
EPY25542.1	MER0870540	metallopeptidase	Metallopeptidase	AAA e Peptidase M41 (Pfam)	(=)	(=)	(=)	(=)	
EPY21849.1	MER0817250	thimet oligopeptidase	Metallopeptidase	Peptidase M3 (Pfam)			(=)	(=)	
EPY37270.1	MER0824176	leishmanolysin	Metallopeptidase	Peptidase M8 (Pfam)	(-)	(+)			
EPY36381.1	MER0824173	leishmanolysin	Metallopeptidase	Peptidase M8 (Pfam)	(-)	(+)			
EPY23056.1		leishmanolysin	Metallopeptidase	Peptidase M8 (Pfam)	(-)	(+)			
EPY20885.1	MER0824394	leishmanolysin	Metallopeptidase	Peptidase M8 (Pfam)	(-)	(+)			
EPY19626.1		leishmanolysin	Metallopeptidase	Peptidase M8 (Pfam)	(-)	(+)			
EPY19508.1		leishmanolysin	Metallopeptidase	Peptidase M8 (Pfam)	(-)	(+)			
EPY32300.1	MER0824163	leishmanolysin-like	Metallopeptidase	Peptidase M8 (Pfam)	(-)	(+)			
EPY21878.1		methionyl aminopeptidase	Metallopeptidase	Peptidase M24 (Pfam)	(-)	(+)			
EPY23120.1	MER0858053	carboxypeptidase Taq	Metallopeptidase	2xPeptidase M32 (Pfam)	(-)	(+)			
EPY22775.1	MER0857611	carboxypeptidase Taq	Metallopeptidase	Peptidase M32 (Pfam)	(=)	(=)	(=)	(=)	

					Brunoro's proteome[10]		Bombaça's proteome[11]		
GenBank ID	MEROPS	Name	Enzymatic class	Domains	WT	APO	WT	APO	Note
EPY23974.1		aminopeptidase	Metallopeptidase	Peptidase M17 (Pfam)	(=)	(=)	(i)	(+++)	
EPY22227.1		aminopeptidase	Metallopeptidase	Peptidase M17 (Pfam)	(=)	(=)			
EPY21441.1	MER0929830	leucyl aminopeptidase	Metallopeptidase	Peptidase M17 (Pfam)	(-)	(+)	(-)	(+)	
EPY15728.1	MER0907441	COP9 signalosome complex subunit 5	Metallopeptidase	JAB_MPN (Smart - Interpro)	(+)	(-)			
EPY35012.1		20S proteasome subunit beta 3	Metallopeptidase	JAB_MPN (Smart - Interpro) e MitMem_reg (Pfam)	(=)	(=)	(=)	(=)	
EPY27615.1		aminopeptidase	Metallopeptidase	Peptidase M24 (Pfam)	(+++)	(i)	(=)	(=)	

WT: wild-type; APO: aposymbiotic; (-): undetected in this strain; (+): exclusively detected in this strain; (i): less abundant expression; (+++): more abundant expression; (=): equal expression; “Blank”: undetected in both strains.
